# Dynamical modeling predicts an inflammation-inducible CXCR7+ B cell precursor with potential implications in lymphoid blockage pathologies

**DOI:** 10.7717/peerj.9902

**Published:** 2020-09-29

**Authors:** Jennifer Enciso, Luis Mendoza, Elena R. Álvarez-Buylla, Rosana Pelayo

**Affiliations:** 1 Centro de Investigación Biomédica de Oriente, Delegación Puebla, Instituto Mexicano del Seguro Social, Metepec, Puebla, Mexico; 2 Centro de Ciencias de la Complejidad, Universidad Nacional Autónoma de México, Mexico City, México; 3 Programa de Doctorado en Ciencias Bioquímicas, Universidad Nacional Autónoma de México, Mexico City, México; 4 Instituto de Investigaciones Biomédicas, Universidad Nacional Autónoma de México, Mexico City, México; 5 Instituto de Ecología, Universidad Nacional Autónoma de México, Mexico City, México

**Keywords:** B cell development, Hematopoietic microenvironment, Bone marrow, Lymphoid niche, Boolean networks, Dynamical modeling, Early lymphoid precursor, Inflammation, NFkB, CXCR7

## Abstract

**Background:**

The blockage at the early B lymphoid cell development pathway within the bone marrow is tightly associated with hematopoietic and immune diseases, where the disruption of basal regulatory networks prevents the continuous replenishment of functional B cells. Dynamic computational models may be instrumental for the comprehensive understanding of mechanisms underlying complex differentiation processes and provide novel prediction/intervention platforms to reinvigorate the system.

**Methods:**

By reconstructing a three-module regulatory network including genetic transcription, intracellular transduction, and microenvironment communication, we have investigated the early B lineage cell fate decisions in normal and pathological settings. The early B cell differentiation network was simulated as a Boolean model and then transformed, using fuzzy logic, to a continuous model. We tested null and overexpression mutants to analyze the emergent behavior of the network. Due to its importance in inflammation, we investigated the effect of NFkB induction at different early B cell differentiation stages.

**Results:**

While the exhaustive synchronous and asynchronous simulation of the early B cell regulatory network (eBCRN) reproduced the configurations of the hematopoietic progenitors and early B lymphoid precursors of the pathway, its simulation as a continuous model with fuzzy logics suggested a transient IL-7R^+^ ProB-to-Pre-B subset expressing pre-BCR and a series of dominant B-cell transcriptional factors. This conspicuous differentiating cell population up-regulated CXCR7 and reduced CXCR4 and FoxO1 expression levels. Strikingly, constant but intermediate NFkB signaling at specific B cell differentiation stages allowed stabilization of an aberrant CXCR7^+^ pre-B *like* phenotype with apparent affinity to proliferative signals, while under constitutive overactivation of NFkB, such cell phenotype was aberrantly exacerbated from the earliest stage of common lymphoid progenitors. Our mutant models revealed an abnormal delay in the BCR assembly upon NFkB activation, concomitant to sustained Flt3 signaling, down-regulation of Ebf1, Irf4 and Pax5 genes transcription, and reduced Ig recombination, pointing to a potential lineage commitment blockage.

**Discussion:**

For the first time, an inducible CXCR7^hi^ B cell precursor endowed with the potential capability of shifting central lymphoid niches, is inferred by computational modeling. Its phenotype is compatible with that of leukemia-initiating cells and might be the foundation that bridges inflammation with blockage-related malignancies and a wide range of immunological diseases. Besides the predicted differentiation impairment, inflammation-inducible phenotypes open the possibility of newly formed niches colonized by the reported precursor. Thus, emergent bone marrow ecosystems are predicted following a pro-inflammatory induction, that may lead to hematopoietic instability associated to blockage pathologies.

## Introduction

Hematopoietic stem cell (HSC) commitment to the many blood cell types takes place in specialized three-dimensional structures known as niches, within bone marrow (BM) microenvironment. Inside the HSC niches, lineage fate decisions are tightly regulated by a large number of molecular and environmental cues secreted by stromal cells, that influence gene transcriptional programs determining cellular specialization. The early stages of B cell differentiation pathway are associated with the functional expression of specific transcription factors, including Ikaros (IKZF1), PU.1, Forkhead Box O1 (FoxO1), early B-cell factor (EBF1), E protein E2A, paired box protein 5 (PAX5), Aiolos (IKZF3) and interferon regulatory factor 4 (IRF4), among others. Their coordinated expression is fundamental for the progressive differentiation of HSCs into multipotent progenitors (MPP), lymphoid-primed multipotent progenitors (LMPP), common lymphoid progenitors (CLP), prepro-B cells, pro-B cells, pre-B cells, and finally immature naive B cells endowed with the ability of migrating to extramedullary secondary lymphoid organs. In turn, each developmental stage is constituted by heterogeneous subpopulations differing in unique cell phenotypes and differentiation potentials, and a common subjacent regulatory network responsive to microenvironmental cues may contribute to the pathway continuum.

During B lymphopoiesis, interleukin-7 (IL-7) is critical for the adequate expression of the pre- and B cell receptors (pre-BCR and BCR, respectively) (Ochiai et al., 2012; Clark et al., 2013; Welner, Pelayo & Kincade, 2008; Bendall et al., 2014). The cytokine IL-7 was originally proposed as a determinant factor for EBF1 induction and BCR recombination in mice (Kikuchi et al., 2008; Roessler et al., 2007), while less inter-dependence in human B lymphopoiesis was observed (Welner, Pelayo & Kincade, 2008). However, more recent research on human B cells harboring IL-7R*α* mutations has shown reduced production of immunoglobulins, suggesting that IL-7 signaling in humans might be compensated by an equivalent but not completely redundant molecular axis that allows cell differentiation in the absence of optimal IL-7 signaling (Nodland et al., 2011; Welner, Pelayo & Kincade, 2008). On the other hand, HSC and CLP populations are highly dependent on CXCL12, a cytokine produced and secreted by BM mesenchymal CAR-type stromal cells (MSC-CAR) (Greenbaum et al., 2013). CXCL12 participates in primitive hematopoietic cells homing, maintenance and retention in BM niches, in part through the regulation of VLA-4/VCAM-1 integrins axis (Peled et al., 2000; Glodek et al., 2003). CXCL12, which binds to CXCR4 expressed in hematopoietic cells, has been also suggested as a crucial regulatory axis in B cell malignancies, like childhood acute lymphoblastic leukemia (ALL) (Greenbaum et al., 2013; Pérez-Saldivar et al., 2011; Sugiyama et al., 2006; Tokoyoda et al., 2004; Enciso et al., 2016; Balandrán et al., 2017).

In addition to its clear dependence on secreted factors, the population dynamics throughout B cell development is crucially supported by specialized stromal niches. Tokoyoda and colleagues have proposed the participation of spatially excluding niches of CXCL12 and IL7 producing stromal cells in the cell expansion/arrest periods of lymphoid precursors to allow the proper V(D)J recombination and further assembly of pre-BCR and BCR, concomitant to a dynamic cell transit from one to another (Tokoyoda et al., 2004; Park et al., 2013; Clark et al., 2013). Though, recent data suggests that IL-7-secreting MSCs are also the highest producers of CXCL12, and that the IL-7/CXCL12 signaling shift depends on IL-7 availability and on the regulation of CXCR4 (Cordeiro Gomes et al., 2016; Fistonich et al., 2018; Zehentmeier & Pereira, 2019).

Biological perturbations of key molecular elements including external factors, might be detrimental to the final balance of regulatory networks involved in cell fate decisions and contribute to progression of hematological malignancies, like ALL. Leukemogenesis appears to be an emergent event of deregulated biological modules involved in signal-transduction, proliferation and apoptosis, genetic regulatory networking and intercommunication with the BM microenvironment. Further convergence of multiple perturbed modules may facilitate aberrant cellular processes occurring in lymphoid niches and the induction of secondary mutations in pre-leukemic clones (Mullighan et al., 2008; Ochodnicka-Mackovicova et al., 2015; Papaemmanuil et al., 2014).

During systemic inflammation, the plasticity of primitive cells leads hematopoiesis to undergo adjustments upon proliferation and cell fate reprogramming in response to stress mediated by proinflammatory factors and recognition of damage- and pathogen-associated molecular patterns via toll-like receptors (TLRs) (Welner, Pelayo & Kincade, 2008; Enciso et al., 2016; Balandrán et al., 2017). The implication of these phenomena in the hematopoietic niches and their supported developing cells on disease progression is research in progress (Terashima et al., 2016; Zehentmeier & Pereira, 2019; Enciso et al., 2016; Lévesque et al., 2003). In addition to ALL, increasing evidence on acute and chronic diseases have shown unfunctional B-lymphopoiesis linked to extrinsic pro-inflammatory pathways leading to intracellular changes in lymphoid progenitors. For example, deficient B cell development in obesity and aging has been related to the adipogenic BM microenvironment containing high levels of pro-myelocytic chemokines and growth factors (i.e., RANTES, G-CSF and GM-CSF) that induce the loss of lymphoid-biased HSCs and inhibit pro-B to pre-B cell transition (Adler et al., 2014; Ambrosi et al., 2017; Riley, 2013). Moreover, during sepsis, the chronic exposure to endogenous (i.e., DAMPS) and exogenous (i.e., viral infection) TLR ligands impairs the CLP capability of differentiating toward B cell precursors, while increasing its potential to produce innate lineage cells (Esplin et al., 2011).

Although CXCL12/CXCR4 axis has been considered the most important player in chemotaxis, support and retention of hematopoietic cells into their BM niches (Sugiyama et al., 2006), MSC isolated from patients suffering hematological diseases have shown dysregulation of CXCL12 and stem cell factor (SCF) concomitant to a pro-inflammatory microenvironment (Colmone et al., 2008; Balandrán et al., 2017; Vilchis-Ordoñez et al., 2015). Accordingly, we have investigated the microenvironment-mediated disruption of lymphopoiesis in pro-inflammatory ALL BM (Balandrán et al. 2017;) and identified by computational modelling CXCR7 as an emergent CXCL12 chemokine receptor playing a potential role in the intercellular communication between hematopoietic precursors and mesenchymal stromal cells (MSC) when intermittent or continuous TLR agonist signals are present (Enciso et al., 2016; Tarnowski et al., 2010). Of high interest, a transient CXCL11^hi^ niche in patient-derived organoids, BM aspirates and biopsies from B-ALL patients, unable to sustain normal lymphopoiesis and endowed with a unique transcriptional signature, has been recently discovered (Balandran et al., 2020, unpublished data). Its ability to attract CXCR3^hi^CXCR7^hi^ leukemic cells even in a CXCL12^low^ scenario favor the hypothesis of CXCR7 as an important player in leukemogenesis (Enciso, Pelayo & Villarreal, 2019).

CXCR7, the atypical chemokine receptor ACKR3, is a seven transmembrane-spanning receptor often co-expressed with CXCR4 by the same cells (Burns et al., 2006; Wang et al., 2018). Since its discovery, the functional activity of CXCR7 as a signaling or non-signaling receptor has not been conclusive. While some findings suggest that it might interact with CXCR4 and work as a decoy receptor, additional evidence demonstrates its capability of signaling through beta-arrestin-2 or G-proteins to induce cellular migration and proliferation (Rajagopal et al., 2010; Levoye et al., 2009; Chen et al., 2015; Odemis et al., 2012; Mazzinghi et al., 2008; Wang et al., 2018). Its dramatic induction prompts a CXCL12 signaling switch from pro-survival pathways (AKT and ERK1/2) to pro-inflammatory pathways (p38 and JNK) (reviewed in Wang et al., 2018). CXCR7 endogenous ligands include CXCL12 (SDF-1), CXCL11/IFN-inducible T cell *α*-chemoattractant (I-TAC, with more affinity than CXCL12), macrophage-inhibitory factor (MIF), adrenomedullin (ADM), bovine adrenal medulla 22 (BAM22) and viral chemokines like vCCL2/vMIP-II (Wang, Cheng & Shen, 2018, Wang et al., 2018). CXCR7 is reported to participate in the induction of monocytes recruitment in allergic inflammation, hematopoietic stem and progenitor cells (HSPC) cycling, as well as B-lymphocyte and tumor cell migration (Alampour-Rajabi et al., 2015; Tarnowski et al., 2010; Torossian et al., 2014; Chang et al., 2018). Of note, CXCR7 has been found increased in ALL cells with a potential role in leukemia progression by recruiting initiating cells to particular BM niches (Melo et al., 2018). Furthermore, increasing evidence of tumor microenvironment controlling CXCR7 expression, particularly in the context of hypoxia, has implicated it as a promising biomarker for anti-tumor and anti-metastatic drug development (reviewed by Wang et al., 2018).

By unraveling the subjacent mechanisms through which local inflammatory processes lead to hematopoietic instability and *de novo* BM niche formation, we may learn about the etiology of lymphoid disorders and also suggest new means to intervene their progression. Here, we propose the reconstruction of three modules (i.g. differentiation genetic core, communication axes with the microenvironment and transduction signaling pathways) merged in a complex early B cell regulatory network (eBCRN) to provide fundamental information on cell population dynamics in the context of pro-inflammatory cues.

Previous work has reconstructed and integrated lymphoid regulatory cores in hematopoiesis network models, including simulation of early lymphoid-myeloid branching, and lymphoid fate decisions toward B, T and NK lineage cells (Collombet et al., 2017; Mendoza & Méndez, 2015; Liquitaya-Montiel & Mendoza, 2018). Nevertheless, information remains scarce about extrinsic mechanisms driving the transition to functional damage of seminal cells or their progeny from a complexity perspective, integrating the microenvironment to which the system is exposed.

The eBCRN was initially simulated as a discrete Boolean model, a methodology useful to represent molecular dynamical descriptions without sufficient kinetic and mechanistic information. As most biochemical signals are continuous in nature (i.e., cytokine/interleukin concentration gradients), models that allow continuous range of activation values to simulate biological systems are, certainly, more appropriate (Enciso, Pelayo & Villarreal, 2019). In order to fulfil this feature, the Boolean rules were transformed to a continuous model using fuzzy logic. By analyzing the model as discrete and continuous dynamical systems, we confirmed its potential to reproduce all early B cell differentiation stages, while by simulating a number of biological perturbations implicating the NFkB pathway, we discovered a putative conspicuous cell population of potential clinical interest. The presented *in silico* tool may contribute to prediction of pre-malignant settings and provide microenvironmental-based strategies to reinvigorate normal hematopoietic pathways.

## Methods

### Boolean dynamical modeling of an early B cell regulatory network (eBCRN)

For the computational modeling of the eBCRN complex system, we followed the standard steps to convert it into a discrete dynamical system, as described by Albert & Wang (2009) and Assman & Albert (2009). The Boolean approach considers that each node of the network can be represented as a binary element, allowed only to have an “active” (ON) or “inactive” (OFF) state, numerically represented by 1 and 0, respectively. The activation state of each node changes over time (measured as iterations or time-steps) according to:

(1) \begin{eqnarray*}& & {X}_{n}(t+1)={f}_{n}({X}_{n1}(t),{X}_{n2}(t),\ldots ,{X}_{nk}(t)),\end{eqnarray*}

where *X*_*n*_(*t* + 1) is the state of the node *X* at time *t* + 1. That state is determined by a Boolean function *f*_*n*_, which depends on the state of the regulators *X*_*n*1_, *X*_*n*2_, …, *X*_*nk*_ at time *t*.

The Boolean function of a particular node, also called logical rule, describes the necessary conditions for its activation, according to the activation state of its regulatory nodes and their interaction. Classical Boolean operators employed for the construction of the Boolean functions are AND, OR and NOT. The AND operator is used to represent the requirement of the conjunction of two or more regulatory nodes; OR operator, when there is more than one node able to regulate another, but only one of them is sufficient to exert the effect; and the NOT operator represents a negative or inhibitory regulation. The Boolean functions were implemented with the BoolNet R package (Müssel, Hopfensitz & Kestler, 2010) to obtain the model attractors after simulation, using the synchronous or asynchronous updating schemes (Albert & Wang, 2009).

In the synchronous model, every node in the network makes a transition at the same time, assuming that all genes and molecules represented in the model take equivalent amount of time in changing their expression or activation levels. This assumption is biologically unrealistic and is addressed through asynchronous simulations, where every single time step one node is randomly selected to be updated, so all nodes are equally likely to make a transition (Garg et al., 2008).

Simulations with both types of updating schemes were run exhaustively, meaning that all the possible initial states were considered for the simulation. Nodes representing single molecules within the network are lower case, while molecular complexes and modules, upper case. Throughout the manuscript, gene symbols are according to conventional nomenclatures in order to distinguish them from network nodes.

### Continuous dynamical simulation of the eBCRN

For transformation to a continuous model, the Boolean rules were submitted to the booleanToODE() function implemented in the BoolNetPerturb R package available at GitHub: https://github.com/mar-esther23/boolnet-perturb. This function transforms the Boolean rules into algebraic expressions based on either Zadeh or probabilistic logics, and then transforms them into a set of ordinary differential equations (ODEs) using either SQUAD (Mendoza & Xenarios, 2006) or the method proposed by Villarreal and colleagues (Villarreal, Padilla-Longoria & Alvarez-Buylla, 2012; Enciso, Pelayo & Villarreal, 2019). For all Boolean-to-continuous transformations, we used Zadeh logics and Villarreal method, where the expression level of *w*_*i*_, representing the Boolean regulatory function for node *i* transformed to an algebraic expression, may be parameterized by a characteristic function with a logistic structure described by the following membership function:

(2) \begin{eqnarray*}& & \Theta [{w}_{i}]= \frac{1}{1+\mathrm{exp}[-b({w}_{i}-{w}_{thr})]} .\end{eqnarray*}

Here, *b* is the saturation rate that indicates the progression of *w*_*i*_ from an unexpressed to an expressed state. This transition is gradual for a small *b* and sharp for a large *b*. We used an intermediate value of *b*, *b* = 10. *w*_*thr*_ represents the threshold between falsity and truth, or in the present context, between inactivation and activation. We set its value to 0.5.

For the continuous simulation, the Boolean rules are replaced in *w*_*i*_, with regulatory functions transformed with Zadeh logics using the following rules:

**Table:** 

Boolean	Zadeh
*q* AND *p*	min [*q, p* ]
*q* OR *p*	max [*q, p* ]
NOT *p*	1-*p*

The dynamic evolution of the expression level *qi(t)* is determined by a set of ODEs of the form:

(3) \begin{eqnarray*}& & \frac{{d}_{qi}}{dt} =\Theta [{w}_{i}]-{\alpha }_{i}{q}_{i},\end{eqnarray*}

where *αi* is the decay rate of the expression of node *i*. We set the parameter *αi* for all nodes equal to 1, so that the expression level of *the node* at its stationary state is merely determined by the degree of truth of the fuzzy proposition *wi*.

For the initial state of simulations, we set the nodes Ikzf1, Gfi1, Spi1 and Runx1 to 1, as they are the required ones for the transition between the HSC and the LMPP attractor. Additionally, we set Flt3L and Il7 to 1, representing a microenvironment with availability to these extrinsic signals. Then, the ODEs system was solved for 100 time-steps, from time 0 to 20 by 0.2.

The dynamic simulation of the differential equations was calculated using the ode() function from the deSolve R package.

### State transition analysis of mutant networks simulated as continuous model

For the validation of the network, we evaluated the effect of all possible single-mutations in the early B lymphopoiesis stages identified with the continuous simulation of our wild-type network. Possible mutations considered for each node were: overactivation (OA), simulated as constitutive value equal to 1; null or knock-out (KO), simulated as totally ablated activation or activation value equal to 0. After deriving each mutant from the wild-type Boolean network, we followed the same methodology to transform and simulate as a continuous model.

After updating the network state from time 0 to 20, separated by 0.2 step, total of 100 time-steps, we calculated the Jaccard index for each time step in the simulations for the wild and mutated networks in order to evaluate the divergence between simulations. The Jaccard index was calculated with the following formula:

(4) \begin{eqnarray*}& & J(W,M)_{t}= \frac{{|}{W}_{t}\bigcap {M}_{t}{|}}{{|}{W}_{t}\bigcup {M}_{t}{|}} = \frac{{|}{W}_{t}\bigcap {M}_{t}{|}}{{|}{W}_{t}{|}+{|}{M}_{t}{|}-{|}{W}_{t}\bigcap {M}_{t}{|}} .\end{eqnarray*}

Here, *W* and *M* represent the wild-type and mutant network configuration at time step *t,* respectively. Network configuration refers to the vector with activation values of all nodes in the network, except the mutated node defining the *M* network. |*W*_*t*_⋂*M*_*t*_| represents the number of nodes with the same activation value at time-step *t* in the continuous simulation of the *W* and the *M* networks, while |*W*_*t*_⋃*M*_*t*_| represent the nodes in each network that had different activation value at time-step *t* in the *W* and the *M* networks simulation. For the Jaccard index calculation, we did not consider the fixed node in the mutated network, which by default would reflect a divergence between the two compared states, *W*_*t*_ and *M*_*t*_, even if the rest of the nodes had identical activation values. Jaccard indexes for all mutants were plotted in a heatmap and the transitions grouped by hierarchical clustering to visualize the common stages where the mutations perturb the normal B cell differentiation process. Code used for model simulation and distance calculation is included in Supplemental Information.

## Results

### A regulatory network controlling early B cell differentiation

**Figure 1:**
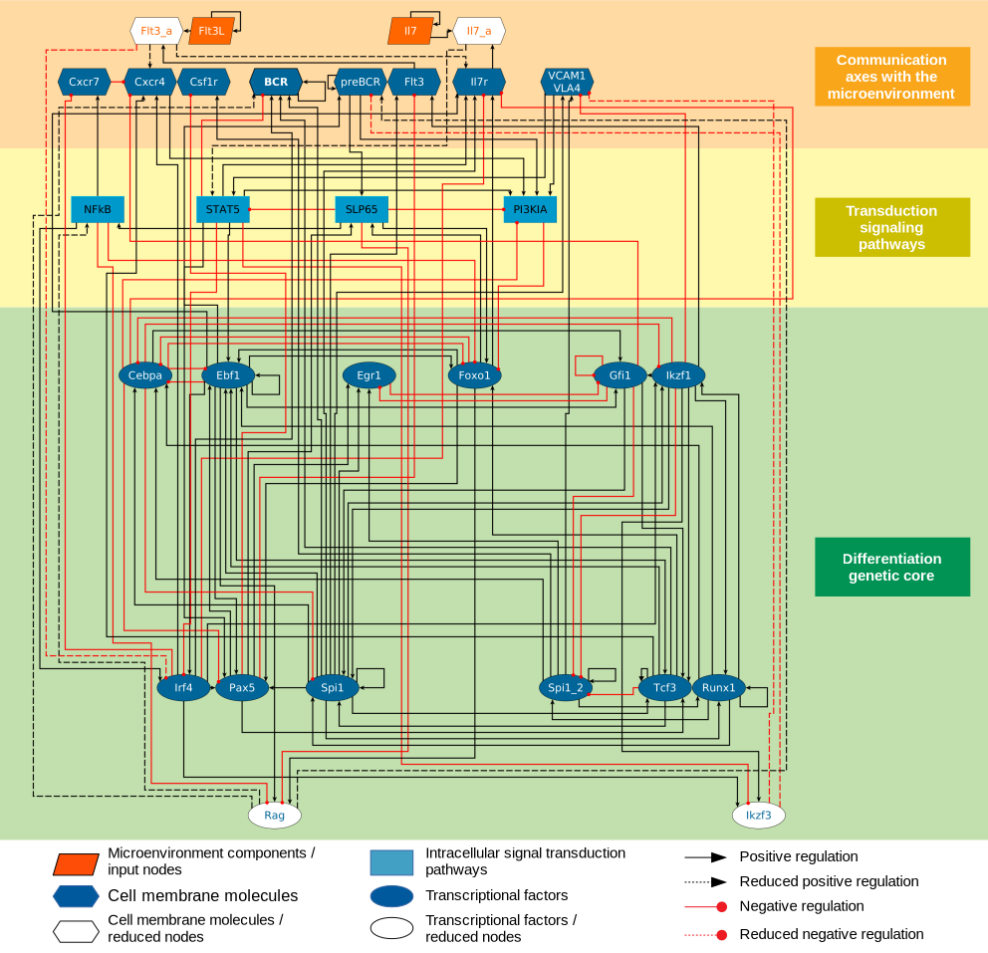
Early B cell regulatory network (eBCRN). The eBCRN was initially composed by 30 nodes, and after reduction, by 26 nodes and 132 interactions. Nodes in white are elements that were reduced from the network. The network includes componentes representing microenvironment secreted molecules, cell membrane receptors, intracellular signal transductors and transcriptional factors. The network diagram was built using yEd Graph Editor (yWorks, https://www.yworks.com).

The proposed eBCRN is grounded on the manual curation of experimental data available in the literature, mainly from murine and human cells. Seed nodes considered to initiate the network reconstruction were crucial transcriptional factors regulating early B lymphopoiesis, including Ikaros, PU.1, FoxO1, EBF1, E2A, Aiolos, PAX5 and IRF4 (Mendoza & Méndez, 2015; Collombet et al., 2017). Ikaros, PU.1 and FoxO1 participate at a very early differentiation stage, during lymphoid/myeloid specification. When highly expressed, PU.1 has been associated to myeloid differentiation, while at intermediate levels induce lymphoid specification through up-regulation of RUNX1 and Ikaros. Its intermediate level is maintained by GFI1 and E2A (Huang et al., 2008; Spooner et al., 2009; Rogers et al., 2016; Zarnegar & Rothenberg, 2012). Here, we considered two independent nodes to simulate the intermediate and high PU.1 expression, represented as Spi1 and Spi1_2 in the network, respectively (Fig. 1). RUNX1 is also an activator of Ikaros, EBF1 and the myeloid transcription factor CEBP/*α*.

During B lineage commitment, the expression of the FLT3 receptor is induced by Ikaros and PU.1. After binding its ligand (FLT3L), FLT3 signaling targets the transcription of IL-7R*α* in cooperation with PU.1 and FoxO1, defining the phenotype of the common lymphoid progenitor (CLP). Downstream the stimulation of IL-7, JAK2 phosphorylates STAT5 and induces their dimerization and translocation into the nucleus, where, by binding their DNA promoter sequences, they activate their target genes EBF1, IRF4 and PAX5. EBF1 activation in pro-B cells completes the set of transcription factors required to induce Recombination Activating 1 and 2 (RAG1/2) expression for the pre-BCR display and the cell transition towards a pre-B cell configuration. The activation of the pre-BCR is not yet clear but it has been suggested that upon locating in lipid rafts, it activates itself by binding *λ*5 chains and/or galectin-1, a component expressed in the BM extracellular matrix (Übelhart et al., 2010; Gauthier et al., 2002). The pre-BCR signaling is represented in the network by the B cell linker protein, BLNK also known as SLP65 or BASH. SLP65 expression is induced by FoxO1 and PAX5, and in turn antagonizes IL-7 signaling. Notably, SLP65 enhances the accumulation of FoxO1 which is down-regulated by IL-7 signaling and consolidates IRF4 expression that induces the transcription of a second zinc finger factor of the Ikaros family, Aiolos (Ochiai et al., 2012).

From previous data reports on Ikaros and Aiolos participation in the reduction of a number of integrins such as VLA-5, CD90 and L-selectin in BM cells and other cell types, we assume that they may also influence negatively the regulation of integrin VLA-4 (Churchman & Mullighan, 2017; Joshi et al., 2014; Li et al., 2014). This assumption is consistent with the decrease in the capacity of pre-B cells to adhere to the VLA-4 receptor, VCAM-1, differentiation stage where expression of Aiolos occurs concomitant to Ikaros (Tokoyoda et al., 2004). The final requisite for pre-B to immature cell transition involves a second wave of RAG1/2 activation downstream the pre-BCR signaling for the recombination and joining of the V and J segments of the light chain. After this step, the BCR of the immature B cells is expressed in the cell membrane. Detailed compiling of reviewed references for the network reconstruction and the development of the logical rules can be found in Table S1.

The inclusion of CXCR4/CXCL12 and VCAM-1/VLA-4 axes is based on their essential role in homeostasis maintenance through the regulation of HSPC migration, engraftment and retention within the BM (Sugiyama et al., 2006; Tzeng et al., 2011; Peled et al., 2000; Ramirez et al., 2009), as well as their particular influence in B lineage support (Ma et al., 1998; Tokoyoda et al., 2004). In our previous model, consisting in a HSPC/MSC intercellular communication network, these molecular axes were integrated with representative downstream signaling pathways and microenvironmental factors involved in the attachment and detachment of hematopoietic cells in mesenchymal BM niches (Enciso et al., 2016). However, for the present model we didn’t incorporate all the molecular elements regulating CXCR/CXCL12 and VLA-4/VCAM-1 expression and activation, as we prioritized on their interactions with crucial B-lymphoid differentiation transcriptional factors. Though, we also conserved the CXCR7 node in order to evaluate its theoretical behavior during early B cell differentiation, and predict if its inflammation-induced expression might affect the production of B lymphocytes in the BM.

Even when there is no direct evidence of CXCR7 activity in early lymphoid progenitor cells CXCR7 promoter contains binding sites for the transcription factor c-Myb, whose conditional deletion in B-cell progenitors blocks the CLP to prepro-B transition (Greig et al., 2010). Furthermore, relevant data from RNA microarray analyses of lympho-hematopietic progenitor cells show a light expression of CXCR7 (GEO accession: GSE64919), which might be maintained in low values until extra medullar B cell maturation (Humpert et al., 2014).

The logical rules of four particular nodes were redirected to their downtream targets in order to reduce from 30 to 26 nodes the eBCRN, keeping its main dynamical properties (Naldi et al., 2011). Boolean rules of the nodes representing the activated IL7R (Il7r_a) and FLT3 (Flt3_a), as well as nodes representing IKZF3 and RAG1/2 (Ikzf3 and Rag), were substituted directly in the Boolean rules of their targets in order to reduce these four nodes from the network (Table S2). The final network used for the computational simulations is composed by 26 nodes (Fig. 1) including two input nodes: Il7 and Flt3; six cell membrane molecules: CXCR4, CXCR7, Flt3, Il7r, preBCR and VCAM1_VLA4; four signal transduction elements: NFkB, STAT5, SLP65 and PI3KIA; 12 transcriptional factors: Cebpa, Ebf1, Egr1, Foxo1, Gfi1, Ikzf1, Irf4, Pax5, Spi1, Spi_2, Tcf3 and Runx1; and two outputs, Csf1r and BCR.

### The eBCRN model reproduces all developmental stages of early B lymphopoiesis

**Figure 2:**
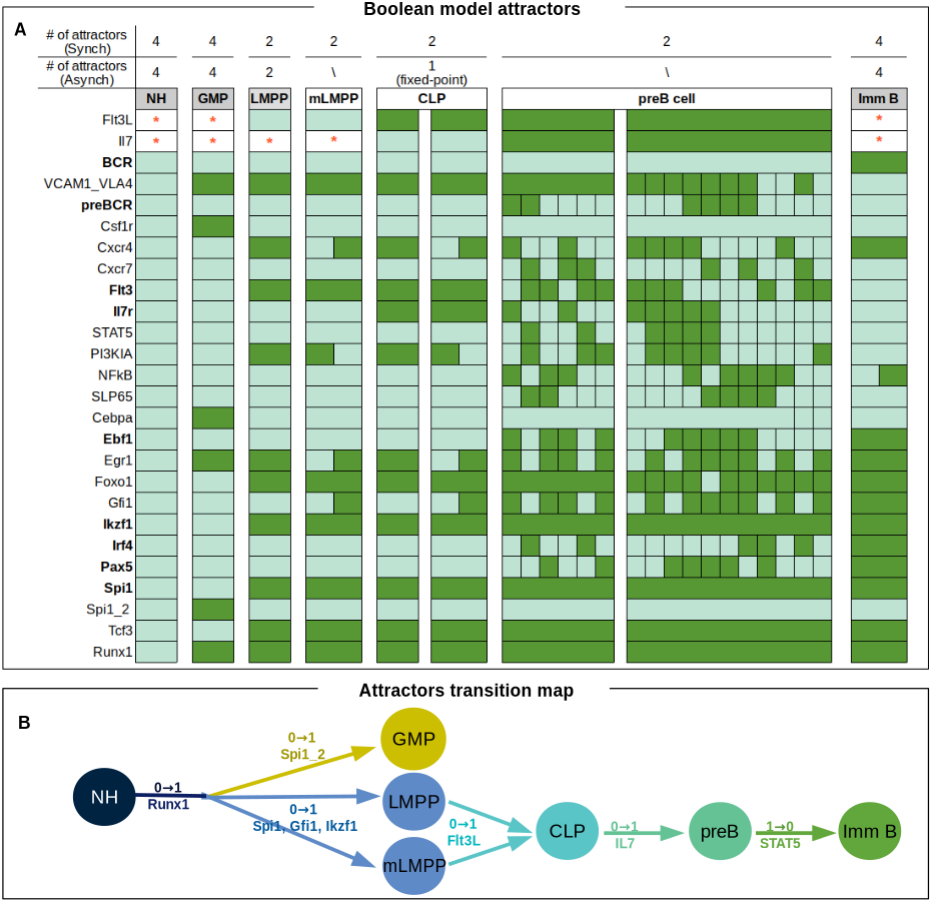
Synchronous and asynchronous attractors recovered from computational simulation of the eBCRN. (A) Attractors labeled according to their compatibility with cellular stages of B lymphopoiesis. Particular lymphoid markers used for this classification are highlighted in bold. The transition between the identified phenotypes resembles in vivo events occuring during B cell differentiation within the bone marrow. (B) Transitions between attractors induced by one-bit flip perturbations (0 to 1, 1 to 0) occur in the same direction as in the differentiation process.

The reconstructed network was exhaustively simulated as a Boolean model using synchronous and asynchronous update schemes until convergence to the system attractors (Fig. 2A). From the synchronous scheme, 20 attractors were recovered. Independently from the input activation state, four fixed-point attractors corresponded to null-attractors, identified in Fig. 2 as non-hematopoietic or NH. The remaining attractors were classified in seven phenotypes according to the configuration of transcription factors and cell membrane molecules. Four fixed-point attractors were classified as granulocyte macrophage progenitors (GMP) as they stabilized with an activated form of nodes representing the Colony Stimulating Factor 1 Receptor (Csf1r), Cebp/*α* (Cebpa), a high level of PU1 (Spi1_2) and no activation of lymphoid lineage markers. The following four attractors correspond to a LMPP phenotype, which is prompt to the lymphoid lineage and characterized by an intermediate level of PU.1 (Spi1), the activation of Ikaros (Ikzf1), FoxO1 (Foxo1), E2A (Tcf3) and particularly FLT3 (Flt3). In the synchronous simulations, we found two types of LMPP attractors diverging on the activation of CXCR4, PI3KIA, Egr1 and Gfi1. Two of these LMPP network states correspond to cyclic attractors that alternate between the activation and the inactivation of the mentioned nodes. Such attractors were named mLMPP due to their compatibility with a migratory state associated to down-regulation of Egr1, which has been related to an increased mobilization of primitive cells in and outside the marrow (Bettini et al., 2002; Min et al., 2008). Interestingly, as a consequence of Egr1 intermittent activation, its target Gfi1 transfer this oscillation to CXCR4, one of the key elements of the network involved in the cellular proximity with stage-specific differentiation BM niches (Cordeiro Gomes et al., 2016). The emergence of the two CLP attractors was dependent on the activation of Flt3L and the absence of IL7 to avoid its progression toward a pre-B cell configuration. The main characteristic of CLP attractors is the expression of the IL-7 receptor (Il7r). Pre-B phenotype was identified for two multi-state attractors by the expression of preBCR and its downstream elements, SLP65 and NFkB, besides the oscillatory activation of late differentiation B cell factors, like PAX5 and IRF4. Finally, 4 fixed-point attractors with stable expression of BCR, Pax5 and Irf4, were associated to an immature B cell phenotype.

Simulations with an asynchronous update strategy reached all the null, myeloid, LMPP and immature attractors, in addition to the fixed-point CLP attractor reproduced with the synchronous updating. To confirm that this network architecture is capable of reproducing the observed biological transitions during early B cell differentiation, we further evaluated transitions between attractors upon one-bit flip perturbations (Fig. 2B). Considering the null attractor as an initial state, the activation of Runx1 in combination with Spi1_2, redirects the network towards the GMP attractor, whereas the lymphoid lineage branch is more restricted and require the activation of Runx1, Spi1, Ikzf1 and Gfi1. LMPP and mLMPP attractors transit to CLP with the activation of Flt3L, which in consequence up-regulates IL7r and, if IL7 is activated, the network progress to a pre-B attractor. To transit from pre-B to an immature B stage, the network requires the down-regulation of STAT5, giving support to accumulated evidence around the importance of down-regulating IL7/STAT5 signaling to continue its differentiation to a B cell phenotype. The mechanism for B cell progenitors to regulate this signal in the BM is still under research.

### Continuous eBCRN modeling reveals intermediate stages of lymphopoietic precursors

**Figure 3:**
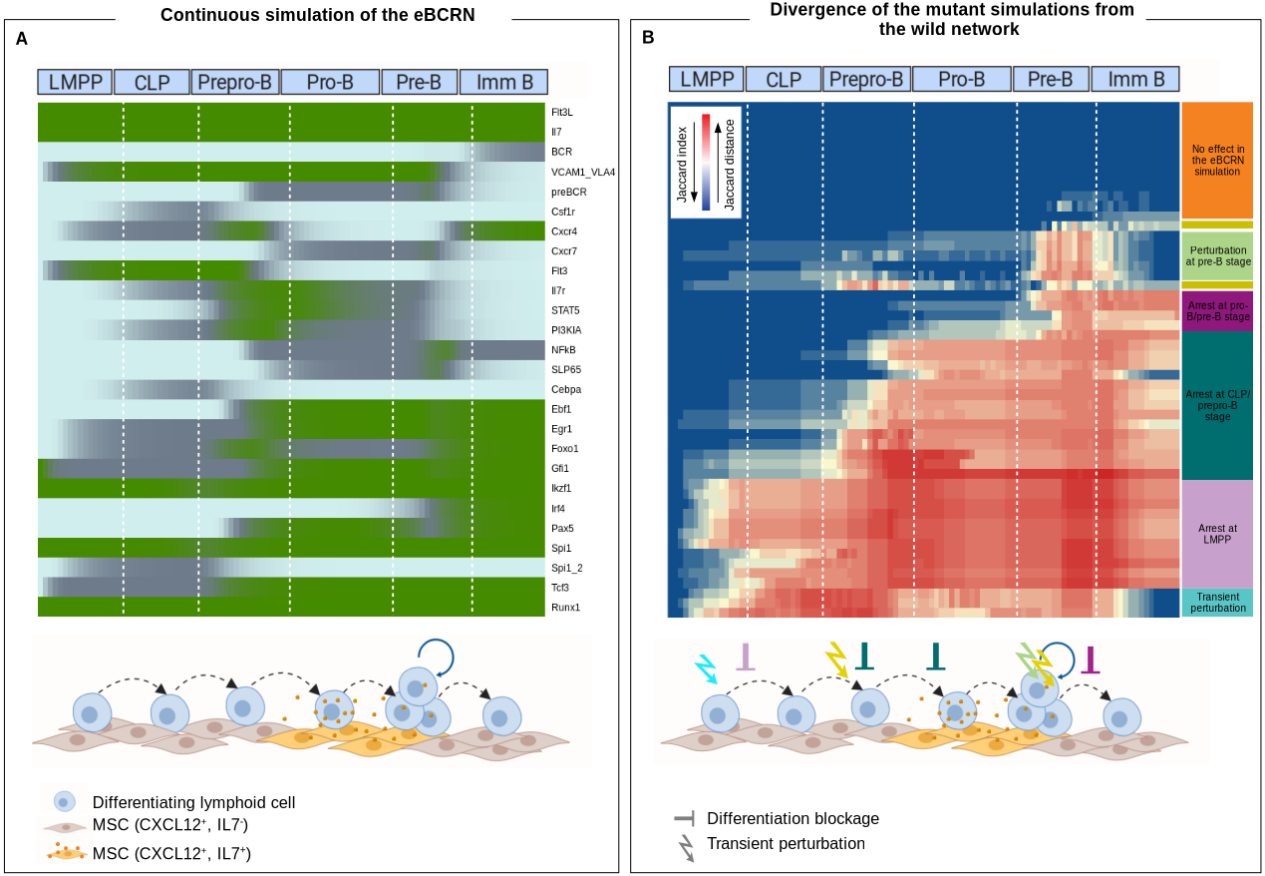
Wild-type and mutant eBCR networks simulated as continuous models. (A) Simulation of the wild-type network. The heatmap shows the activation value for each node of the eBCRN simulated for 100 time-steps. The illustration represents cell transitions in the BM inferred by the activation of microenvironment communication molecules modulated during the continuous simulation. (B) Mutant effects on the early B cell differentiation. The heatmap shows the distance per time-step (columns) calculated using Jaccard index, between each mutant (rows) and the wild-type network. The mutant simulations cluster depends on the developmental stage where simulation diverge from the wild-type network. The developmental stages that are affected by each cluster are depicted.

Because cell differentiation is not dependent on fully discrete decisions, in order to reproduce intermediate or transient states, we transformed the model from discrete to a continuous dynamical system. The continuous simulation of the eBCRN allowed the display of transitions not previously evidenced by the Boolean model. To compute the continuous model simulation, we used as initial state the minimum network configuration necessary for the transition from HSC to LMPP, which implicated the activation of Ikzf1, Gfi1, Spi1 and Runx1. HSC transitions to the immature B cell stable state in the context of microenvironment (depicted by activated Flt3L and Il7), are represented in Fig. 3A. As expected, the continuous simulation reproduced the gradual regulation of key molecular components, as the myeloid lineage factor Cebpa, whose expression is recorded in LMPPs and CLPs as a residual granulocytic potential marker. Cebpa lowers its expression gradually, in parallel to the progression towards prepro-B and pro-B stages (Guo et al., 2018; Karamitros et al., 2018). In pre-B stage, the continuous simulation of the eBCRN model evidences the increase and subsequent modulation of SLP65 and NFkB participating downstream activation of the preBCR, which in turn is down-regulated upon the Irf4 node activation. Of note, the transitory activation of NFkB pathway during lymphopoiesis, has been associated to a negative feedback loop involved in the regulation of RAG activity to stabilize the DNA recombination events (Ochodnicka-Mackovicova et al., 2015).

Regarding the expression of membrane receptors involved in the BM spatial transition of progenitors in response to IL7 and CXCL12 gradients, we identified five major stages: IL7r^+^CXCR4^high^, IL7r^high^CXCR4^−^, IL7r^low^CXCR4^low^ and IL7r^−^CXCR4^high^. Based on previous evidence of CXCR7 up-regulation by NFkB signaling activation, this alternative receptor for CXCL12 may contribute the observed CXCR4 down-regulation, to specifically induce B lymphoid progenitors migration across BM mesenchymal niches (Uto-Konomi et al., 2013; Zabel et al., 2009; Melo et al., 2018), considering that CXCR7 affinity for CXCL12 is 10-fold higher than for CXCR4 (Hartmann et al., 2008; Balabanian et al., 2005). Of special interest, by replicating the BM microenvironment in leukemia patient-derived organoids (PDO), our recent findings have revealed a CXCL11^+^ hematopoietic niche in childhood leukemia that display a unique signature and support leukemia initiating cells (Balandrán, 2019-2020, unpublished data). Such CXCL11^+^ specialized niche, absent in normal conditions, might be presumably colonized by the CXCR7^+^ blasts found in ALL (Jalili et al., 2008; Melo et al., 2018). Whether the abnormal B precursor cells induced by inflammation and inferred for the first time in this report, are positioned in inducible CXCL11^+^ niches before triggering the first steps of hyperproliferative or malignant diseases, is a crucial question to address.

### Mutant eBCRN networks and the emergence of aberrant lymphoid blockage phenotypes

To validate the eBCRN network, we generated all possible mutant networks and simulated their transition from the LMPP attractor, excluding the mutated node. We then compared the divergence between each of the 52 mutant simulations and the wild-type landscape by calculating a Jaccard index at each time-step. The results are represented in a heatmap reordered with hierarchical clustering to group the mutations that affect the same developmental stage (Fig. 3B). The detailed experimental evidence for each mutation in every cluster is referenced in Table S3.

With exception of three networks, the effect of every mutated network simulated as a continuous model occurred at almost the same developmental stage as reported in the available literature. The three exceptions include networks with Flt3 mutations, KO and OA, and the network with OA of Egr1. The experimental evidence for the inhibition and the constitutive expression of Flt3 indicates that perturbation in lymphoid development occurs in earlier stages than what is suggested by the simulations. This discrepancy may result from the Flt3/Flt3L downstream signaling pathway considered, as we included this molecular axis as one of the various driving factors for the expression of IL7R. Interestingly, the simulation of Egr1 OA turned out in a complete blockage of B cell development at LMPP stage, indicating that increased expression of Egr1 affects primitive LT-HSC and participate in the development of different types of leukemia (Tian et al., 2016).

The reproduction of the experimental effect on most simulated mutants, strengthen some of the assumptions made during the network construction. In particular, the assumption of the concomitant activation of the VCAM-1/VLA-4 integrin axis as a requirement for activation of the STAT pathway during B cell differentiation, is supported by the experimental evidence of pre-B and mature B cell reduction in chimeric mice with low expression of *α*4, a subunit of VLA-4 integrin (Arroyo et al., 1996). The computational simulation of the VCAM1_VLA4 KO network showed a blockage around the prepro-B stage. Empty of the B cell progenitor compartment by the absence of VCAM-1/VLA-4 and consequently, the reduced STAT activation, could be occurring in parallel to the early release of progenitor cells from the BM, as reported by Ulyanova upon ablation of VCAM-1 in mice fibroblasts (Ulyanova et al., 2005). Further insights in VLA-4 co-signaling activation would help in the understanding of relapse and drug resistance in hematological cancer (Redondo-Muñoz et al., 2010; Shalapour et al., 2011).

Mutant network simulations also provide information about the influence of perturbations on the transcriptional differentiation core. The mutations can be clustered in (i) those that completely block differentiation at different stages and (ii) those that perturb the transition but converge to immature B cell. Among the latter group, we found nodes representing signaling pathways (i.e., PI3KIA OA and KO, Cxcr4 OA and KO, Cxcr7 OA, and NFkB KO) and other related to the main differentiation transcriptional core (i.e., Tcf3 OA, Gfi1 OA, and Ebf1 OA). Tcf3, Gfi1 and Ebf1 have been reported to participate in cell proliferation and apoptosis, suggesting that when up-regulated, they can be involved in this biological activity besides the transcriptional regulation of lymphoid genes.

### A dysfunctional CXCR7^+^ B cell precursor is induced by NFkB activation: the prediction from dynamical modeling

**Figure 4:**
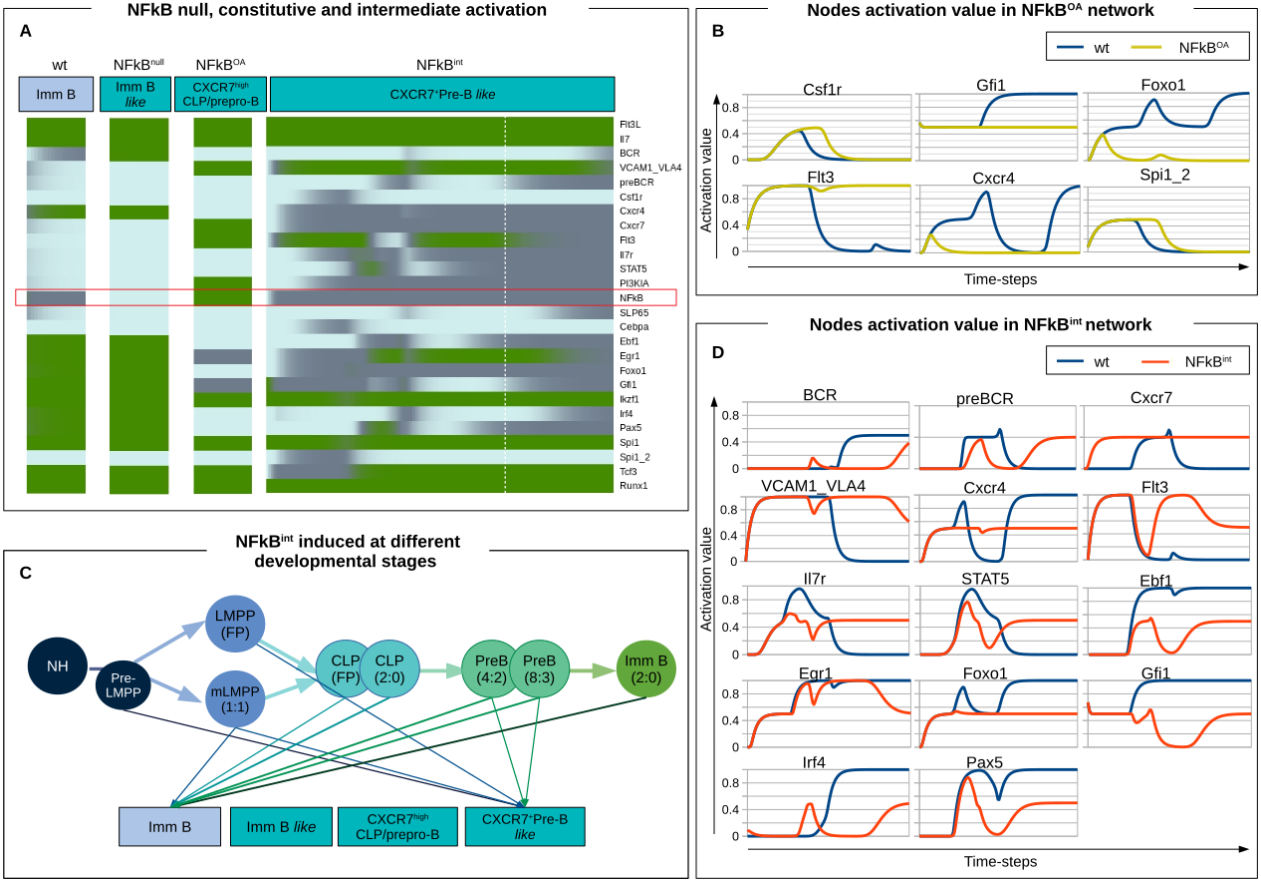
NFκB constitutive activation induces the arrest of aberrant phenotypes in the B cell differentiation pathway. (A) Continuous simulation of NFkB eBCR mutant networks including node knock-out (NFkB = 0) and two levels of constitutive activation (OA, overactivation: NFkB = 1, and int, intermediate: NFkB = 0.5). (B) and (D) Representative nodes in NFkB OA and int mutated eBCRN, respectively. (C) Induction of an intermediate value of NFkB at different developmental stages derive in different attractors, some remaining unperturbed and reaching the immature B cell phenotype, and some arresting the simulation at an aberrant pre-B like phenotype. Above the developmental stage where the NFkB intermediate value was induced (i.e., LMPP, CLP, PreB and Imm B), are indicated the number of stages of the cyclic attractors reaching the immature B (Imm B) stage and the number of stages redirecting the simulation to an aberrant pre-B *like* phenotype (CXCR7^+^Pre-B *like*). FP: Fixed point attractor.

We have previously reported that pro-inflammatory molecules are directly involved in the disruption of conventional communication axes between stroma and hematopoietic cells, contributing to shape a pathological onco-promoting microenvironment (Vilchis-Ordoñez et al., 2015; Balandrán et al., 2017; Enciso et al., 2016). Accordingly, this work aimed to a deeper analysis of NFkB perturbations. As expected, the NFkB KO network converged, after 20 time-steps, to the same attractor as the wild-type network, while the OA of NFkB resulted in an attractor arrested between the CLP and the prepro-B phenotype. The mixed CLP/prepro-B attractor displayed a higher expression level of Flt3, and an intermediate activation of Gfi1 and Egr1 as in CLP stage, but down-regulated expression of Cebpa, like the found in prepro-B stage (Fig. 4A). The OA of NFkB crucially inhibits Foxo1, a factor indispensable for Il7r expression explaining the blockage at this stage. After analyzing the transition from the initial state to the differentiation blockage, we found that the myeloid markers Csfr1 and Spi1_2 extended their activation in comparison with the normal transition, while lymphoid nodes like Gfi1 and Foxo1 were down-regulated (Fig. 4B). This is particularly interesting because it has been reported that TLR ligands, through canonical Myd88/NFkB signaling, are capable of re-directing BM hematopoiesis toward a myeloid fate concomitant to changes in cell fate decisions and migration of lymphoid progenitors to extramedullary sites (Nagai et al., 2006).

Chronic inflammation plays a supportive role in oncogenesis by increasing cellular vulnerability to DNA damage and epigenetic changes that affect tumor immune surveillance and predispose to clonal evolution. The establishment of chronic pro-inflammatory feedback loops inducing microenvironment remodeling and/or cellular damage accumulation, might occur through different mechanisms, including the activation of inflammasome and TLRs by endogenous ligands (i.e., intestinal bacteria-derived liposaccharide, senescence-associated secretory phenotype cytokines, miRNAs and danger-associated molecular pattern molecules) (Ratajczak et al., 2018; Franceschi et al., 2018; Fabbri et al., 2012; Hernandez, Huebener & Schwabe, 2016).

Nevertheless, the role of a progressive and chronic inflammatory effect on the development of lymphoproliferative diseases, like ALL, is not fully understood. While in acute inflammation we may think in an all-or-non phenomena -as in the KO and OA simulations-, our hypothesis is that the continuum pro-inflammatory process involved in a positive feedback loop between leukemic and stromal cells, is smooth. To computationally test this hypothesis, we modified the logical rule for NFkB, replacing its logical rule with a negative self-regulation (NFkB = !NFkB) that induces the constitutive intermediate activation of the node (Fig. 4A). This condition produced a delayed pre-B-like phenotype around the 40th time-step, while all previous simulations got to their attractors between time-steps 20 and 30. Unlike its pre-B conventional counterpart, the pre-B like attractor expresses intermediate values for nodes Egr1, Ebf1, Gfi1 and Pax5. When analyzed the intermediate stages (Fig. 4D) we noticed that the simulation occurs similarly to the *wt* network simulation and apparently reaches a transitory pre-B state, although some nodes are unable to reach normal activation levels, as is the case of Cxcr4, Il7r, STAT5, Ebf1, Foxo1 and Gfi1. To evaluate whether the alternative attractor generated by the intermediate activation of NFkB depends on the differentiation state at which the perturbation was induced, we simulated the NFkB^int^ network beginning at multiple differentiation stages. We ran simulations by setting as initial states each of the single state attractors and the states within the cyclic attractors found in the Boolean synchronous simulation (Fig. 4C). Only eight simulations out of 26, converged in the pre-B like attractor, which included the LMPP single state attractor, one of the two states of the cyclic mLMPP attractor, and 5 states of the pre-B cyclic attractors. None of the two network states in the CLP cyclic attractor were susceptible to the intermediate activation of NFkB.

Thus, the detrimental effect of NFkB perturbation in progenitor lymphoid cells is dependent on its activation level and the differentiation stage in which the perturbation occurs. NFkB influences the hematopoietic-microenvironment communication axes, by inhibiting CXCR4, upregulating CXCR7 and maintaining an increased level of VCAM-1. Additionally, it directly affects the function of transcription factors involved in the differentiation process and stabilize abnormal phenotypes leading to differentiating cell arrest.

We suggest, for the first time, that the chronic or acute proinflammatory environment is capable of initiating pathological processes in the lympho-hematopoietic differentiation pathway, by inducing the emergence of dysfunctional populations with potential ability of colonizing non-conventional niches.

## Discussion

By modeling the normal B cell developmental pathway in the bone marrow, we have explored potential microenvironmental elements cooperating in the earliest stages of leukemogenesis, and identified a unique differentiating subpopulation as a potential target for CXCR7 up-regulation induced by pro-inflammatory signals, that may confer the ability of migrating to alternate niches. Our predictions agree with recent notions suggesting the highly dynamic communication between developing cells and flexible niches during pathological processes. Computational tools are useful for studying plasticity properties of subjacent regulatory modules (i.e., transcriptional, intercellular communication, signal transduction, cell cycle, etc.), for visualizing transient phenotypes and for predicting potential outcomes in abnormal settings. Here, we reconstructed a regulatory network that merges transcription factors regulating early B lymphopoiesis with signaling pathways involved in the communication with BM stromal microenvironments, to test the interconnectivity of canonical signaling pathways mediating the cellular response to the microenvironment, with particular focus on the NFkB pro-inflammatory pathway.

The simulation of the network as a Boolean model was capable of reproducing the activation states of the LMPP, CLP, preB cell and immature B cell within the pathway. As a continuous model, additional patterns were identified: prepro-B and pro-B.

**Figure 5:**
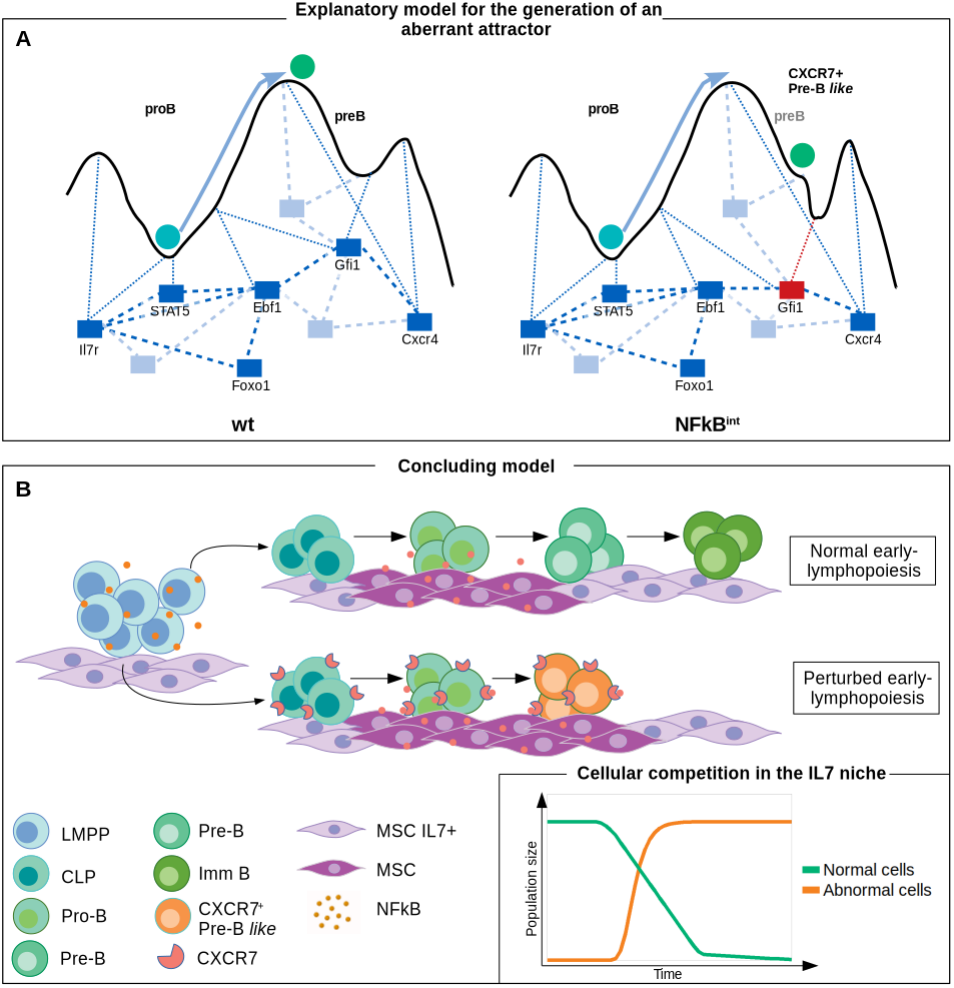
NF*κ*B activation induces a perturbation in the early B cell differentiation regulatory network promoting the potential development of an aberrant B cell precursor: a prediction from dynamical modeling. (A) Network perturbations affect the epigenetic landscape and may derive in de novo aberrant attractors. (B) Both, normal and aberrant (i.e. malignant) hematopoietic cells production originate from similar progenitors. However, the aberrant population induced by a pro-inflammatory derived NFkB signal is capable of arresting the path at a precursor-like stage with distinct niche-transition abilities. The affinity of the aberrant population to particular BM niches, suggested by the expression of molecules involved in microenvironment communication axes, may represent an ecological advantage for the progressive replacement of normal hematopoiesis observed in leukemic malignancies.

Most mutant simulations reproduced the effects reported in the literature, particularly for those that induce a complete blockage in a differentiation stage. Mutations which did not induce a significant effect in the final stable state (i.g. mutant networks reproducing immature phenotypes and inducing only temporal perturbations) include nodes that mostly influence processes other than transcriptional regulation. Such set of mutations shows their greater distance, expressed as high Jaccard indexes in Fig. 3B, to the wild-type pre-B stage, a differentiation stage characterized for a vigorous clonal expansion and a selective outgrowth of pre-BCR-expressing cells. Even though the transcriptional network is not affected, their perturbation may influence processes like proliferation, recombination and clonal selection (Benhamou et al., 2018). Upon simulation of the continuous model with NFkB constitutive, null and intermediate activation, we infer that NFkB is dispensable for B cell differentiation. However, its high activation induces an aberrant attractor and a longer transitory stage with myeloid potential, redirecting differentiation toward a non-lymphoid lineage, as previously reported in ALL murine models (Cheung et al., 2018). Moreover, a conspicuous population with a pre-B *like* phenotype and particular niche requirements developed upon the intermediate activation of NFkB, emerging as a perturbation in the epigenetic landscape (Fig. 5A). An interesting consequence of intermediate NFkB constitutive expression is the observed downregulation of FOXO1, suggesting NFkB-mediated inflammation as an alternative or synergic mechanism for the induction of RAG1 overexpression in B-ALL. The induction of the intermediate expression of FOXO1 can promote tumor proliferation and survival in ALL cell lines (Alkhatib et al., 2012; Wang et al., 2018).

According to the notion implicating a pivotal role of IL7 and CXCL12 niches in lymphoid developmental process, we propose that the abnormal pre-B *like* cells expressing CXCR7 and IL7 receptor, correspond to a cellular subset able to stay in the CXCL12^+^IL7^+^ niche, where they might outcompete and displace normal pre-B cells, affecting the production of immature B cells (Fig. 5B). The replacement of normal hematopoiesis has been suggested to occur during leukemic progression through different mechanisms, including niche occupancy by hyperproliferative cells and induction of a leukemic microenvironment unsuitable for normal hematopoiesis (Balandrán et al., 2017; Cheung et al., 2018). In a previous work, we reported the NFkB-induced expression of CXCR7 concomitant to the perturbation of the CXCR4/CXCL12 and VCAM-1/VLA-4 integrin axes (Enciso et al., 2016), consistent with experimental evidence of its involvement in immune cell recruitment and tumor metastasis (Alampour-Rajabi et al., 2015; Tarnowski et al., 2010; Torossian et al., 2014; Chang et al., 2018). However, despite CXCR7 reported overexpression in leukemic cells (Melo et al., 2018), there were no studies on CXCR7 regulation along normal and pathological BM lymphoid differentiation. One of the most relevant predictions of the present work is the sustained expression of CXCR7 by an abnormal inflammation-induced pre-B *like* phenotype, that may endow them to displace normal lymphopoietic precursors from CXCL12 niches due to the 10-fold higher binding affinity of CXCR7, compared to CXCR4 (Balabanian et al., 2005).

As mentioned, CXCR7 has been extensively documented in the pathobiology of an increasing number of systemic diseases, including inflammation (rheumatoid arthritis, obesity), cancer (prostate, colon, bladder, ovarian, cervical, uterine, kidney, liver, stomach, lung, breast and hepatocelular), neurological conditions, infectious and other complex diseases, like autism and atherogenesis (reviewed in Lounsbury, 2020). Although for all of them destabilization of the immune system is recorded, it is uncertain whether the participation of pro-inflammatory elements is accompanied by agonist signals provided by DAMPs, PAMPs and possibly LAMPs (Zindel & Kubes, 2020), and if central mechanisms are activated within the bone marrow that change the fate of developing cells or shift the identity of lymphoid niches. Investigating its occurrence as a generic mechanism that influence lineage decisions and potentially cooperates with other ontogeny regulation systems (Martínez-Barnetche et al., 2002), would be of crucial relevance for the understanding, comprehensive management or prevention of general inflammatory conditions.

This emphasize the substantial need of promoting feedback loops between experimental and computational research to investigate the potential participation of CXCR7 in migration/homing of early precursors to unique activating microenvironments in lymphoid-related pathologies where inflammation functions as a cooperative element, such as acute leukemia, bone marrow failure, acute and chronic viral infections, sepsis, metabolic and autoimmune diseases, among others.

Of high interest, by using patient-derived organoids to reconstruct BM microenvironment from ALL patients, we have recently identified a transient CXCL11^hi^ niche in B cell acute lymphoblastic leukemia (ALL) patients, unable to sustain normal lymphopoiesis and endowed with a unique transcriptional signature (Balandran et al., 2020, unpublished data). Its ability to attract CXCR3^hi^CXCR7^hi^ leukemic cells even in a CXCL12^low^ scenario favor the hypothesis of CXCR7 as an important player in disease. To validate our computational model predictions, CXCR7 expressing cells should be investigated, both in normal and inflammation settings, using in vitro and ex vivo experimental approaches, or in NSG xenotransplantation pre-clinical models. Upon high purification, fully characterized cells must be studied for their preference to colonize special microenvironments.

Together, this and other pieces of work suggest that pro-inflammatory signals lead to a number of developmental outcomes, including the production of normal immature B cells or arrested pre-B like cells, depending on the stage at which the stimulus occur. Thus, the complex configuration of the activated transcriptional factors and signaling pathways may define the cell vulnerability to perturbation (Rodríguez-Hernández et al., 2019; Espinoza-Sanchez et al., 2018).

## Conclusions

Currently, a linear map for the lymphoid developmental pathway has been depicted in depth with respect to transcriptional programs regulating the progressive loss of multipotential cell differentiation concomitant to gain of functional commitment, in strict dependence on growth factors. However, in the setting of systemic diseases related to inflammation, special mechanisms come into play and skew normal differentiation patterns to restore homeostasis, with the latent risk of harming developing tissues.

The integration of genetic and epigenetic microenvironmental elements orchestrating complex biological ecosystems has required additional efforts to understand such emergent diseases that depend on the interconnection of molecular networks and the cellular interaction with its microenvironmental surrounding. Our mathematical model may give a glance in the importance of alternative chemotactic receptors, as CXCR7, responding not only to CXCL12 but also to CXCL11 and additional chemokines within the BM and at extramedullary tissues like liver, spleen, and lung. Thus, novel molecular axes must be further considered as inducible pieces of the complex epigenetic landscape in transitional stages and disease.

##  Supplemental Information

10.7717/peerj.9902/supp-1 Table S1 Bibliographic justification for all interactions comprising the eBCRN and its corresponding Boolean rules upon manual curation Boolean operators are represented by symbols & (AND), | (OR) and ! (NOT).

10.7717/peerj.9902/supp-2 Table S2 Logical rules used for computational simulation of the eBCRN The logical rules of the reduced components were replaced in their target nodes. The reduced nodes and replaced logical rules are identified by gray color. Boolean operators are represented with the symbols & (AND), | (OR) and ! (NOT).

10.7717/peerj.9902/supp-3 Table S3 Extended results and analyses from the continuous simulations of all possible mutant networks KO, Knock out; OA, overactivation.

10.7717/peerj.9902/supp-4 Supplemental Information 1 R Code is used for simulation of the Boolean network, its transformation to continuous model, for the continuous simulation and distance calculation between network states implementing Jaccard Index

10.7717/peerj.9902/supp-5 Supplemental Information 2 Boolean network rules

## Additional Information and Declarations

### Data Availability

The following information was supplied regarding data availability: The code is available in the Supplemental Files.
