# Supplementary material for: Dynamical modeling predicts an inflammation-inducible CXCR7+ B cell precursor with potential implications in lymphoid blockage pathologies
Source: PeerJ. 2020 Sep 29;8:e9902. doi: 10.7717/peerj.9902 (PMC7531334; doi:10.7717/peerj.9902)
Supplement: Table S1 — Boolean operators are represented by symbols & (AND), | (OR) and ! (NOT). [file peerj-08-9902-s001.doc]

**Supplementary Table 1.** Bibliographic justification for all interactions comprising the eBCRN and its corresponding Boolean rules upon manual curation. Boolean operators are represented by symbols & (AND), | (OR) and ! (NOT).

| **Node** | **Boolean rule** | **Interaction** | | **Observation** | **Reference** |
| --- | --- | --- | --- | --- | --- |
| Flt3L | Flt3L | Input | | | |
| Il7 | Il7 | Input | | | |
| BCR | Spi1 & Tcf3 & Irf4 & !STAT5 & (Rag | BCR) | Spi1 | + | Spi1, Irf4, and Tcf3 bind to the κE3′ intronic enhancer. Removal of the TCF3 binding sites impairs recombination by Rag. | (Perfetti et al., 2004; Ma et al., 2006; de Pooter & Kee, 2010; Hodawadekar et al., 2012) |
|  |  | Tcf3 | + |  |  |
|  |  | Irf4 | + |  |  |
|  |  | Rag | + | RAG is reactivated during the pre-B stage to participate light chain (IgL) recombination. |  |
|  |  | STAT5 | - | STAT5 maintains the the Igκ locus repressed at the pro-B cell stage, inhibiting Tcf3 binding. |  |
|  |  | **BCR *** | + | BCR self-regulation replaces the single Igκ recombination event promoted by Rag that determines the transition from pre-BCR to BCR. | *Assumption |
| VCAM1_VLA4 | (Spi1 | Spi1_2) & !(Ikzf1 & Ikzf3) | Spi1 | Spi1_2 | + | HPCs mutated in PU.1 lack integrins alpha-4, subunit of VLA-4. | (Welner, Pelayo & Kincade, 2008; Ochiai et al., 2012; Clark et al., 2013; Bendall et al., 2014) |
|  |  | **Ikzf1 & Ikzf3 *** | - | Ikaros and Aiolos promote pro-B cell migration through the downregulation of adhesins. Because pre-B cells express both factors and have increased migration toward CXCL12 without adhering via VCAM-1, we propose VLA4 as another Ikzf1/Ikzf3 target. | *Assumption (Mullighan et al., 2008; Papaemmanuil et al., 2014; Ochodnicka-Mackovicova et al., 2015) |
| preBCR | Ebf1 & !Ikzf3 & (Rag | preBCR) | Ebf1 | + | Binding sites for EBF can be found in the genes for λ5 and VpreB, participating in the assembly of pre-BCR. | (Ochiai et al., 2012) |
|  |  | Ikzf3 | - | Aiolos is a negative modulator of λ5, subunit of the pre-BCR surrogated light chain. | (Li et al., 2014; Joshi et al., 2014; Churchman & Mullighan, 2017) |
|  |  | Rag | + | RAG1 and RAG2 initiate Ig heavy chain (Igh) recombination. | (Tokoyoda et al., 2004) |
|  |  | preBCR | + | Pre-BCR self-regulation replaces the transient activation of Rag. In vivo, pre-BCR self-induction of activation in cooperation with its binding to stromal ligands like galectin-1. | (Bettini et al., 2002; Min et al., 2008) |
| Csf1r | Spi1_2 & !Pax5 | Spi1_2 | + | PAX5 interferes with PU.1 transactivation at the sense promoter of Csf1r gene and down regulates the frequency of binding of the basal transcription machinery. | (Cordeiro Gomes et al., 2016) |
|  |  | Pax5 | - |  |  |
| Cxcr4 | Tcf3 & (!Gfi1 | Flt3_a | Irf4) & !Cxcr7 | Tcf3 | + | CXCR4 expression was down-regulated in a transcriptional profiling of E-protein–deficient activated B cells. Evaluated E-proteins include E2A and E2-2. | (Karamitros et al., 2018; Guo et al., 2018) |
|  |  | Gfi1 | - | In myeloid cells it has been reported an inhibitory interaction of Gfi1 after binding to Cxcr4 promoter. Additionally, HSCs from Gfi1ko/ko mice show an increased expression of CXCR4. | (Ochodnicka-Mackovicova et al., 2015) |
|  |  | Flt3_a | + | Flt3/Ras-dependent signals suppresses SOCS3, which in turn, negatively regulate CXCR4. | (Li et al., 2010) |
|  |  | Irf4 | + | IRF4 directly induces the expression of Cxcr4. | (Johnson et al., 2008) |
|  |  | Cxcr7 | - | Overexpression of CXCR7 inhibits CXCR4 expression, besides acting as as an alternative receptor for CXCL12 with higher affintity than CXCR4. | (Uto-Konomi et al., 2013; Coggins et al., 2014) |
| Cxcr7 | NFkB & !Irf4 | NFkB | + | Cxcr7 promoter contains three binding sites to NFkB with probed inhibitor functionallity. | (Tarnowski, Kucia & Ratajczak, 2009; Tarnowski et al., 2010) |
|  |  | Irf4 | - | CXCR7 expresion is up-regulated in IRF4-deficient B cells, controling their positioning in lymphoid microenvironments. | (Simonetti et al., 2013) |
| Flt3 | Ikzf1 & Spi1 & !Pax5 | Ikzf1 | + | Hematopoietic progenitors with inhibited expression of Ikaros or PU.1 are deficient in expression of Flt3. | (Nichogiannopoulou et al., 1999) |
|  |  | Spi1 | + |  | (DeKoter, Lee & Singh, 2002) |
|  |  | Pax5 | - | Progenitor B cells defficient in Pax1, show an abundant expression of Flt3, effect that is rapidly inversed upon the reinduction of Pax5 expression. | (Holmes et al., 2006; Ochiai et al., 2012) |
| Il7r | Spi1 & Foxo1 & (Flt3_a | (STAT5 & Ebf1)) & !(Cebpa | Irf4) | Spi1 | + | PU.1 directly regulates the transcription of the gene coding for the IL-7Rα-chain. | (DeKoter, Lee & Singh, 2002) |
|  |  | Foxo1 | + | Foxo1 expression is required at early B cell developmental stages to sustain the expression of Il7ra. | (Dengler et al., 2008; Ochiai et al., 2012) |
|  |  | Flt3_a | + | Flt3 activation promote the expression of IL-7Rα and suppresses SOCS genes that contribute to the inhibition of STAT5 signaling. | (Li et al., 2010) |
|  |  | STAT5 | + | EBF participates in a feedback loop that sustains and augments IL7r expression after initial activation. | (Singh, Pongubala & Medina, 2007; Pongubala et al., 2008) |
|  |  | Ebf1 | + |  |  |
|  |  | Cebpa | - | ChIP-seq analysis revealed binding of C/EBPα at the cis-regulatory elements of Foxo1, Ebf1, Pax5, IL7r, and Mef2c genes, with inhibitory activity. | (Collombet et al., 2017) |
|  |  | Irf4 | - | Irf4 attenuates Il7r signaling by inducing Cxcr4 expression, promoting the migration of B progenitor cells towards CXCL12, whihch is expressed in a separated niche than the one compossed by IL7-secreting stromal cells. | (Tokoyoda et al., 2004; Johnson et al., 2008) |
| STAT5 | (Il7&Il7r) & VCAM1_VLA4 & !SLP65 | Il7&Il7r | + | IL-7R signaling activates STAT5 and participates in the promotion of distal VH gene rearrangements. | (Bertolino et al., 2005) |
|  |  | **VCAM1_VLA4 *** | + | In endothelial cells, STAT5 pathway activation by IL3 requires the concomitant activation of JAK/STAT by β integrin, VLA4 subunit. We assume that a similar requirement may be involved in the expansion of B cell progenitors in IL7-enrriched bone marrow niches. | *Assumption for hematopoietic cells (Tokoyoda et al., 2004; Defilippi et al., 2005) |
|  |  | SLP65 | - | Adaptor SLP65, also known as BLNK or BASH, inhibits JAK3/STAT5 signaling pathway through its binding to JAK3. | (Nakayama et al., 2008) |
| PI3KIA | (preBCR | (VCAM1_VLA4 & Cxcr4) | Cxcr7) & !SLP65 & (!Cebpa | STAT5) | preBCR | + | Independently of IL-7 signalling, pre-BCR stimulation induces the phosphorylation of AKT in human pre-B cells. | (Anbazhagan et al., 2013) |
|  |  | SLP65 | - | Activation of an inducible form of SLP65 results in markedly reduced levels of phos-phorylated Akt/PKB, downstream phosporylation target of PI3KIA. | (Herzog et al., 2008) |
|  |  | VCAM1_VLA4 | + | Downstream VLA4 binding to VCAM1, ILK induces the phosphorylation of Akt at Ser473 and GSK3b at Ser9. | (Tabe et al., 2007) |
|  |  | Cxcr4 | + | Inhibition of either CXCR4 or CXCR7, derives in decreased activation of PI3K pathway by β-arrestin in CD34+ hematopoietic cells. | (Chabanon et al., 2008; Torossian et al., 2014) |
|  |  | Cxcr7 | + |  |  |
|  |  | Cebpa | - | Cebpa promotes the expression of miR-29b which in turn induces PTEN transcription, an inhibitor for PI3K pathway. | (Eyholzer et al., 2010; Wang et al., 2015) |
|  |  | STAT5 | + | The abundance of phosphorylated Akt was positively correlated with that of IL-7R in mice pre-B cells. | (Ochiai et al., 2012) |
| NFkB | SLP65 | Rag | SLP65 | + | After pre-BCR incorporates into cell membrane lipid rafts it derives in the activation of a complex molecular module composed by Lyn, Syk, SLP65, PI3K, Btk, Vav, and PLCγ2. The activation of PLCγ2 induces calcium signaling and subsequently NFkB activation. | (Schebesta, Pfeffer & Busslinger, 2002; Kersseboom et al., 2003) |
|  |  | Rag | + | Rag activity during V(D)J recombination, induce an ATM-dependent DNA damage response. Among other functions, ATM kinase phosphorylates IKK releasing the NFkB complex. | (Ochodnicka-Mackovicova et al., 2016; Meek et al., 2016) |
| SLP65 | preBCR & (Foxo1 | Pax5) | preBCR | + | BLNK transduce pre-BCR activation upon phosphorylation by Syk. | (Pappu et al., 1999; Kersseboom et al., 2003) |
|  |  | Foxo1 | + | Foxo1 in conjunction with Pax5, activates SLP65 coding gene expression in pre-B cells enabling an effective coupling of the pre-BCR with its downstream signaling components. | (Ochiai et al., 2012) |
|  |  | Pax5 | + | Pax5 upregulates the expression of Blnk gene, involved in the transduction of the pre-BCR activation. | (Schebesta, Pfeffer & Busslinger, 2002; Ochiai et al., 2012) |
| Cebpa | (Spi1 | Runx1) & (!(Ebf1 | Foxo1 | Ikzf1) | (Runx1 & Spi1_2)) | Runx1 | + | Runx1 gene deletion reduces Cebpa transcripts through binding to sites in the promoter region. | (Guo et al., 2012) |
|  |  | Spi1 | + | PU.1 inhibits GATA-1 that targets, as an inhibitor, Cebpa required for myeloid development. | (Burda et al., 2009) |
|  |  | Spi1_2 | + |  |  |
|  |  | Foxo1 | - | ChIP-seq experiments probed the binding of FoxO1 to the Cebpa locus. A functional analysis revealed a negative regulation. | (Collombet et al., 2017) |
|  |  | Ebf1 | - | EBF1 and Ikaros bind to silencing regions in the Cebpa gene, having a probable redundant inhibitory role on Cebpa transcription. | (Rao et al., 2013; Bertolino, Reinitz & Manu, 2016) |
|  |  | Ikzf1 | - |  |  |
| Ebf1 | Tcf3 & Runx1 & ((STAT5 & Foxo1) | (Spi1 & Ebf1 & Pax5)) & !Cebpa | Tcf3 | + | E2A is required for initiating and maintaining the expression of EBF to ensure the B cell program specification. | (Kwon et al., 2008) |
|  |  | Runx1 | + | Runx1-binding to motifs in the proximal promoter of Ebf1 are essential to drive gene expression through the regulation of repressive histone marks. | (Seo et al., 2012) |
|  |  | STAT5 | + | The distal promoter of Ebf1 is controlled by IL7R signaling, E2A and EBF1. While the proximal promoter is upregulated by the binding of Pax5, Ets1 and PU.1. | (Roessler et al., 2007; Zandi et al., 2008) |
|  |  | Pax5 | + |  |  |
|  |  | Spi1 | + |  |  |
|  |  | Ebf1 | + |  |  |
|  |  | Foxo1 | + | Increased expression of Foxo1 in combination with IL7R signaling, activate the transcription of the Ebf1 gene. In turn, Ebf1 activates the expression of FoxO1 forming a positive feedback loop. | (Mansson et al., 2012; Katerndahl et al., 2017) |
|  |  | Cebpa | - | ChIP-seq analysis revealed binding of C/EBPα at the cis-regulatory elements of Foxo1, Ebf1, Pax5, IL7r, and Mef2c genes, with an inhibitory activity. | (Collombet et al., 2017) |
| Egr1 | (Spi1 & !Gfi1) | Spi1_2 | Pax5 | Spi1 | + | Egr1 expression is promoted with increased PU.1 levels and attenuated with Gfi1 overexpression. | (Laslo et al., 2006) |
|  |  | Spi1_2 | + |  |  |
|  |  | Gfi1 | - |  |  |
|  |  | Pax5 | + | The induced expression of Pax2/5/8 promotes the upregulation of EGR1, in addition to B-cell related genes. | (Hart et al., 2018) |
| Foxo1 | (Tcf3 | Ebf1) & (!PI3KIA | !NFkB | SLP65) & !Cebpa | Tcf3 | + | E2A binds to enhancer elements in the FoxO1 locus to activate Foxo1 expression. | (Welinder et al., 2011) |
|  |  | Ebf1 | + | Increased expression of Foxo1 in combination with IL7R signaling activate the transcription of the Ebf1 gene. In turn, Ebf1 activates the expression of FoxO1 in a positive feedback loop. | (Mansson et al., 2012) |
|  |  | PI3KIA | - | IL-7 signaling positively correlates with the abundance of phosphorylated Akt, and negatively correlates with the overall abundance of Foxo1 and Foxo3a proteins. Akt phosphorylates Foxo proteins and promote their degradation via ubiquitination. | (Ochiai et al., 2012) |
|  |  | NFkB | - | The treatment of Abl mouse pre-B cells and two human BCR-ABL-positive B-ALL cells with an IKKb inhibitior, negatively regulate FoxO1 stability. | (Ochodnicka-Mackovicova et al., 2015) |
|  |  | SLP65 | + | SLP65 activates p38 which in turn stimulates FoxO1 activity. Establishing a FoxO1-SPL65-p38 feedbackloop. | (Ochiai et al., 2012) |
|  |  | Cebpa | - | ChIP-seq analysis revealed binding of C/EBPα at the cis-regulatory elements of Foxo1, Ebf1, Pax5, IL7r, and Mef2c genes, with an inhibitory activity. | (Collombet et al., 2017) |
| Gfi1 | ((Ikzf1 | Cebpa) &  (!(Egr1 | Gfi1) | Ebf1) | Ikzf1 | + | Ikaros, a transcription factor required for B cell development, promoted Gfi1 and antagonized PU.1 expression in MPPs. | (Spooner et al., 2009) |
|  |  | Cebpa | + | C/EBPα interacts with a functional binding site in the Gfi gene and enhances Gfi-1 expression. | (Lidonnici et al., 2010) |
|  |  | Egr1 | - | Several putative Egr binding sites were identified in the promoter region of the Gfi-1 gene, one of them is bound with high affinity by Egr-1. | (Laslo et al., 2006) |
|  |  | Gfi1 | - | Their have been identified Gfi1 binding motifs conserved among rat, mice and human Gfi1 genes, suggesting a repressive self-regulation mechanism. | (Doan et al., 2004; Yücel et al., 2004; Marteijn et al., 2007) |
|  |  | Ebf1 | + | Predicted promoter/enhancer for GFI1 gene | GeneHancer (GH) Regulatory Elements for GFI1 Gene(Fishilevich et al., 2017) |
| Ikzf1 | (Runx1 & Spi1 & !Cebpa) | Irf4 | Runx1 | + | Runx binding to E3 ligase CRBN inhibitis its activity as promoter of Ikaros ubiquitylation and proteasome-dependent degradation. | (Zhou et al., 2019) |
|  |  | Spi1 | + | PU.1 indirectly induce Ikaros expression through Mef2c transcription factors. Mef2c is inhibited by Cebpa. | (Stehling-Sun et al., 2009; Herglotz et al., 2016) |
|  |  | Cebpa | - |  |  |
|  |  | Irf4 | + | Irf4 participates in the activation of Ikaros and Aiolos to down-regulate pre-B-cell receptor and promote cell-cycle withdrawal in pre-B-cell development. | (Ma et al., 2006, 2008; Thompson et al., 2007) |
| Ikzf3 | Ikzf1 & Irf4 & !STAT5 | Ikzf1 | + | Ikaros binds the Aiolos promoter, inducing its expression in cell lines and freshly isolated B and T cells. | (Ghadiri et al., 2007) |
|  |  | STAT5 | - | STAT5 binds to a large subset of genes that regulate normal progenitor B cell development, including IRF4 and AIOLOS. | (Katerndahl et al., 2017) |
|  |  | Irf4 | + | Irf4 participates in the activation of Ikaros and Aiolos to down-regulate pre-B-cell receptor and promote cell-cycle withdrawal in pre-B-cell development. | (Thompson et al., 2007; Ma et al., 2008) |
| Irf4 | (NFkB | Ebf1) & !((Flt3 & Flt3L) | STAT5) | NFkB | + | C-Rel, a member of the NFkB transcription factors, induces IRF4 expression in lymphocytes. | (Grumont & Gerondakis, 2000) |
|  |  | Ebf1 | + | Ebf1 directly binds to IRF4, IRF8, Myb, Mybl2, and Bcl2l1 sequences, shown by chromatin immunoprecipitation (ChIP) analysis. | (Györy et al., 2012) |
|  |  | Flt3_a | - | Inhibition of Flt3 over the transcription factor IRF4 represents the inhibition of the plasmacytoid cell program in CLP cells. This inhibition may be mediated by STAT5, as reported for IRF8. | (Esashi et al., 2008; Mendoza & Méndez, 2015) |
|  |  | STAT5 | - | STAT5 binds to a large subset of genes that regulate normal progenitor B cell development, including IRF4 and AIOLOS. | (Katerndahl et al., 2017) |
| Pax5 | Spi1 & (STAT5 | (Ebf1 & Foxo1 & Irf4)) & !Cebpa | Spi1 | + | PU.1 binds to an enhancer element in Pax5 gene. While, IRF4 at an intermediate level correlates with nuclear Foxo1 and Pax5 induction. PU.1 binding to an enhancer regulatory element in intron 5 of Pax5. | (Decker et al., 2009; Lu et al., 2014) |
|  |  | Foxo1 | + |  |  |
|  |  | Irf4 | + |  |  |
|  |  | STAT5 | + | STAT5 directly up-regulates Pax5 transcription in early B cells through its binding to a motif in the gene promoter region that overlaps with an EBF-binding site. | (O’Riordan & Grosschedl, 1999; Hirokawa et al., 2003; Decker et al., 2009) |
|  |  | Ebf1 | + |  |  |
|  |  | Cebpa | - | ChIP-seq analysis revealed binding of C/EBPα with an inhibitory activity, at the cis-regulatory elements of Pax5, among other B cell promoting genes. | (Collombet et al., 2017) |
| Rag | Ebf1 & Foxo1 & !(SLP65 | NFkB) | Ebf1 | + | Binding sites for EBF are found in a large number of B cell-specific genes including Rag1. | (O’Riordan & Grosschedl, 1999) |
|  |  | Foxo1 | + | FoxO1 induces Ikzf1 splicing to promote immunoglobulin gene recombination through RAG. | (Alkhatib et al., 2012) |
|  |  | SLP65 | - | Evidence for a direct repression of RAG1 and RAG2 by pre-BCR signaling. | (Schebesta, Pfeffer & Busslinger, 2002) |
|  |  | NFkB | - | Pre-B cells transduced with the IkBa-super represor increases RAG activity. Additionally, IKKβ induces the expression of cyclin A/CDK2 that in its active form, restrict RAG2 expression in cycling cells. | (Ochodnicka-Mackovicova et al., 2015) |
| Spi1 | Runx1 & ((Gfi1 | Tcf3) | (Spi1 & Ikzf1)) & !Cebpa | Runx1 | + | RUNX1 binds to an upstream regulatory element of Spi1. | (Roessler et al., 2007; Kikuchi et al., 2008) |
|  |  | Gfi1 | + | Gfi1 constrains the expression of PU.1 by displacing PU.1 from positive autoregulatory elements. | (Welner, Pelayo & Kincade, 2008) |
|  |  | Spi1 | + |  |  |
|  |  | Tcf3 | + | E2A directly binds to PU.1 antagonizing its activity and inhibiting myeloid differentiation. | (Welner, Pelayo & Kincade, 2008; Nodland et al., 2011) |
|  |  | Ikzf1 | + | Ikaros, which is required for B-cell development, might constrain PU.1 expression by blocking PU.1 autoregulation through the URE. | (Greenbaum et al., 2013) |
|  |  | Cebpa | - | Cebp/α induction of Pu.1 expression is required during myeloid differentiation, characterized by a high level expression of PU.1. | (Peled et al., 2000; Glodek et al., 2003) |
| Spi1_2 | Runx1 & (!(Gfi1 | Tcf3) | (Spi1_2 & !Ikzf1)) | Runx1 | + | RUNX1 binds to an upstream regulatory element of Spi1. | (Tokoyoda et al., 2004; Sugiyama et al., 2006; Pérez-Saldivar et al., 2011; Greenbaum et al., 2013; Balandrán et al., 2016; Enciso et al., 2016) |
|  |  | Gfi1 | - | Gfi1 constrains the expression of PU.1 by displacing PU.1 from positive autoregulatory elements. | (Tokoyoda et al., 2004; Park et al., 2013; Clark et al., 2013) |
|  |  | Spi1_2 | + |  |  |
|  |  | Tcf3 | - | E2A directly binds to PU.1 antagonizing its activity and inhibiting myeloid differentiation. | (Cordeiro Gomes et al., 2016; Fistonich et al., 2018; Zehentmeier & Pereira, 2019) |
|  |  | Ikzf1 | - | Ikaros constrains PU.1 expression by blocking PU.1 autoregulation through the URE. | (Lévesque et al., 2003; Enciso et al., 2016; Terashima et al., 2016; Zehentmeier & Pereira, 2019) |
| Tcf3 | (Ikzf1 | Spi1 | Pax5) & (!Tcf3 | Ebf1 | Gfi1) | Ikzf1 | + | Ikaros knockout results in loss of lymphocytes as seen with E2a knockout. Additionally, Ikaros-binding sites were identified at the E2a promoter in B cells | (Assman & Albert, 2009; Albert & Wang, 2009) |
|  |  | Spi1 | + | Physical interaction observed throug ChIP-seq meta-analysis. | (Müssel, Hopfensitz & Kestler, 2010) |
|  |  | Pax5 | + | Physical interaction observed throug ChIP-seq meta-analysis. | (Albert & Wang, 2009) |
|  |  | Tcf3 | - | E47 binds to the promoter region of Id3, an E2A inhibitory factor. | (Mendoza & Xenarios, 2006) |
|  |  | Ebf1 | + | EBF1 and Gfi1 inhibit ID2, an inhibitor of E2A expression and critial for specification towards the B cell lineage. | (Villarreal, Padilla-Longoria & Alvarez-Buylla, 2012) |
|  |  | Gfi1 | + |  |  |
| Runx1 | (Spi1 | Spi1_2) & (Runx1 | Ikzf1) | Spi1 | Spi1_2 | + | RUNX1 functionally binds to upstream regulatory element of PU.1 regulating its expression. However, PU.1 induced expression is not sufficient to rescue ablation of RUNX1. | (Mendoza & Méndez, 2015; Collombet et al., 2017) |
|  |  | Runx1 | + | RUNX1 regulates its own expression through binding sites in the distal promoter P1 region of Runx1 gene. | (Huang et al., 2008; Spooner et al., 2009; Zarnegar & Rothenberg, 2012; Rogers et al., 2016) |
|  |  | Ikzf1 | + | Ikaros consensus binding sites were found in the proximal promoter regions of the Runx1 gene. | (Gauthier et al., 2002; Übelhart et al., 2010) |

**References**

Albert R, Wang R-S. 2009. Discrete dynamic modeling of cellular signaling networks. *Methods in enzymology* 467:281–306. DOI: 10.1016/S0076-6879(09)67011-7.

Alkhatib A, Werner M, Hug E, Herzog S, Eschbach C, Faraidun H, Köhler F, Wossning T, Jumaa H. 2012. FoxO1 induces Ikaros splicing to promote immunoglobulin gene recombination. *The Journal of experimental medicine* 209:395–406. DOI: 10.1084/jem.20110216.

Anbazhagan K, Rabbind Singh A, Isabelle P, Stella I, Céline A-DM, Bissac E, Bertrand B, Rémy N, Naomi T, Vincent F, Rochette J, Lassoued K. 2013. Human pre-B cell receptor signal transduction: evidence for distinct roles of PI3kinase and MAP-kinase signalling pathways. *Immunity, inflammation and disease* 1:26–36. DOI: 10.1002/iid3.4.

Assman SM, Albert R. 2009. Discrete dynamic modelling with asynchronous update or, how to model complex systems in the absence of quantitative information. *Methods in molecular biology* 553:207–225. DOI: 10.1007/1-4020-0613-6_16551.

Balandrán JC, Purizaca J, Enciso J, Dozal D, Sandoval A, Jiménez-Hernández E, et al. 2016. Pro-inflammatory-Related Loss of CXCL12 Niche Promotes Acute Lymphoblastic Leukemic Progression at the Expense of Normal Lymphopoiesis. *Frontiers in immunology* 7:666. DOI: 10.3389/fimmu.2016.00666.

Bendall SC, Davis KL, Amir ED, Tadmor MD, Simonds EF, Chen TJ, et al. 2014. Single-Cell Trajectory Detection Uncovers Progression and Regulatory Coordination in Human B Cell Development. *Cell* 157:714–725. DOI: 10.1016/j.cell.2014.04.005.

Bertolino E, Reddy K, Medina KL, Parganas E, Ihle J, Singh H. 2005. Regulation of interleukin 7–dependent immunoglobulin heavy-chain variable gene rearrangements by transcription factor STAT5. *Nature Immunology* 6:836–843. DOI: 10.1038/ni1226.

Bertolino E, Reinitz J, Manu. 2016. The analysis of novel distal Cebpa enhancers and silencers using a transcriptional model reveals the complex regulatory logic of hematopoietic lineage specification. *Developmental biology* 413:128–44. DOI: 10.1016/j.ydbio.2016.02.030.

Bettini M, Xi H, Milbrandt J, Kersh GJ. 2002. Thymocyte Development in Early Growth Response Gene 1-Deficient Mice. *The Journal of Immunology* 169:1713–1720. DOI: 10.4049/jimmunol.169.4.1713.

Burda P, Curik N, Kokavec J, Basova P, Mikulenkova D, Skoultchi AI, Zavadil J, Stopka T. 2009. PU.1 Activation Relieves GATA-1-Mediated Repression of Cebpa and Cbfb during Leukemia Differentiation. *Molecular Cancer Research* 7:1693–1703. DOI: 10.1158/1541-7786.MCR-09-0031.

Chabanon A, Desterke C, Rodenburger E, Clay D, Guerton B, Boutin L, et al. 2008. A cross-talk between stromal cell-derived factor-1 and transforming growth factor-beta controls the quiescence/cycling switch of CD34(+) progenitors through FoxO3 and mammalian target of rapamycin. *Stem cells* 26:3150–3161. DOI: 10.1634/stemcells.2008-0219.

Churchman ML, Mullighan CG. 2017. Ikaros: Exploiting and targeting the hematopoietic stem cell niche in B-progenitor acute lymphoblastic leukemia. *Experimental Hematology* 46:1–8. DOI: 10.1016/j.exphem.2016.11.002.

Clark MR, Mandal M, Ochiai K, Singh H. 2013. Orchestrating B cell lymphopoiesis through interplay of IL-7 receptor and pre-B cell receptor signalling. *Nature Reviews Immunology* 14:69–80. DOI: 10.1038/nri3570.

Coggins NL, Trakimas D, Chang SL, Ehrlich A, Ray P, Luker KE, Linderman JJ, Luker GD. 2014. CXCR7 controls competition for recruitment of β-arrestin 2 in cells expressing both CXCR4 and CXCR7. *PloS one* 9:e98328. DOI: 10.1371/journal.pone.0098328.

Collombet S, van Oevelen C, Sardina Ortega JL, Abou-Jaoudé W, Di Stefano B, Thomas-Chollier M, Graf T, Thieffry D. 2017. Logical modeling of lymphoid and myeloid cell specification and transdifferentiation. *Proceedings of the National Academy of Sciences of the United States of America* 114:5792–5799. DOI: 10.1073/pnas.1610622114.

Cordeiro Gomes A, Hara T, Lim VY, Herndler-Brandstetter D, Nevius E, Sugiyama T, Tani-Ichi S, Schlenner S, Richie E, Rodewald H-R, Flavell RA, Nagasawa T, Ikuta K, Pereira JP. 2016. Hematopoietic Stem Cell Niches Produce Lineage-Instructive Signals to Control Multipotent Progenitor Differentiation. *Immunity*. DOI: 10.1016/j.immuni.2016.11.004.

Decker T, Pasca di Magliano M, McManus S, Sun Q, Bonifer C, Tagoh H, Busslinger M. 2009. Stepwise Activation of Enhancer and Promoter Regions of the B Cell Commitment Gene Pax5 in Early Lymphopoiesis. *Immunity* 30:508–520. DOI: 10.1016/j.immuni.2009.01.012.

Defilippi P, Rosso A, Dentelli P, Calvi C, Garbarino G, Tarone G, Pegoraro L, Brizzi MF. 2005. {beta}1 Integrin and IL-3R coordinately regulate STAT5 activation and anchorage-dependent proliferation. *The Journal of cell biology* 168:1099–108. DOI: 10.1083/jcb.200405116.

DeKoter RP, Lee H-J, Singh H. 2002. PU.1 regulates expression of the interleukin-7 receptor in lymphoid progenitors. *Immunity* 16:297–309. DOI: 10.1016/S1074-7613(02)00269-8.

Dengler HS, Baracho G V, Omori SA, Bruckner S, Arden KC, Castrillon DH, DePinho RA, Rickert RC. 2008. Distinct functions for the transcription factor Foxo1 at various stages of B cell differentiation. *Nature immunology* 9:1388–98. DOI: 10.1038/ni.1667.

Doan LL, Porter SD, Duan Z, Flubacher MM, Montoya D, Tsichlis PN, Horwitz M, Gilks CB, Grimes HL. 2004. Targeted transcriptional repression of Gfi1 by GFI1 and GFI1B in lymphoid cells. *Nucleic acids research* 32:2508–2519. DOI: 10.1093/nar/gkh570.

Enciso J, Mayani H, Mendoza L, Pelayo R. 2016. Modeling the Pro-inflammatory Tumor Microenvironment in Acute Lymphoblastic Leukemia Predicts a Breakdown of Hematopoietic-Mesenchymal Communication Networks. *Frontiers in physiology* 7:349. DOI: 10.3389/fphys.2016.00349.

Esashi E, Wang Y-H, Perng O, Qin X-F, Liu Y-J, Watowich SS. 2008. The signal transducer STAT5 inhibits plasmacytoid dendritic cell development by suppressing transcription factor IRF8. *Immunity* 28:509–20. DOI: 10.1016/j.immuni.2008.02.013.

Eyholzer M, Schmid S, Wilkens L, Mueller BU, Pabst T. 2010. The tumour-suppressive miR-29a/b1 cluster is regulated by CEBPA and blocked in human AML. *British journal of cancer* 103:275–84. DOI: 10.1038/sj.bjc.6605751.

Fishilevich S, Nudel R, Rappaport N, Hadar R, Plaschkes I, Iny Stein T, Rosen N, Kohn A, Twik M, Safran M, Lancet D, Cohen D. 2017. GeneHancer: genome-wide integration of enhancers and target genes in GeneCards. *Database : the journal of biological databases and curation* 2017. DOI: 10.1093/database/bax028.

Fistonich C, Zehentmeier S, Bednarski JJ, Miao R, Schjerven H, Sleckman BP, Pereira JP. 2018. Cell circuits between B cell progenitors and IL-7 ^+^ mesenchymal progenitor cells control B cell development. *The Journal of Experimental Medicine* 215:2586–2599. DOI: 10.1084/jem.20180778.

Gauthier L, Rossi B, Roux F, Termine E, Schiff C. 2002. Galectin-1 is a stromal cell ligand of the pre-B cell receptor (BCR) implicated in synapse formation between pre-B and stromal cells and in pre-BCR triggering. *Proceedings of the National Academy of Sciences of the United States of America* 99:13014–9. DOI: 10.1073/pnas.202323999.

Ghadiri A, Duhamel M, Fleischer A, Reimann A, Dessauge F, Rebollo A. 2007. Critical function of Ikaros in controlling Aiolos gene expression. *FEBS Letters* 581:1605–1616. DOI: 10.1016/j.febslet.2007.03.025.

Glodek AM, Honczarenko M, Le Y, Campbell JJ, Silberstein LE. 2003. Sustained Activation of Cell Adhesion Is a Differentially Regulated Process in B Lymphopoiesis. *The Journal of experimental medicine* 197:461–473. DOI: 10.1084/jem.20021477.

Greenbaum A, Hsu Y-MS, Day RB, Schuettpelz LG, Christopher MJ, Borgerding JN, Nagasawa T, Link DC. 2013. CXCL12 in early mesenchymal progenitors is required for haematopoietic stem-cell maintenance. *Nature* 495:227–30. DOI: 10.1038/nature11926.

Grumont RJ, Gerondakis S. 2000. Rel induces interferon regulatory factor 4 (IRF-4) expression in lymphocytes: modulation of interferon-regulated gene expression by rel/nuclear factor kappaB. *The Journal of experimental medicine* 191:1281–92.

Guo H, Barberi T, Suresh R, Friedman AD. 2018. Progression from the Common Lymphoid Progenitor to B/Myeloid PreproB and ProB Precursors during B Lymphopoiesis Requires C/EBPα. *The Journal of Immunology* 201:1692–1704. DOI: 10.4049/jimmunol.1800244.

Guo H, Ma O, Speck NA, Friedman AD. 2012. Runx1 deletion or dominant inhibition reduces Cebpa transcription via conserved promoter and distal enhancer sites to favor monopoiesis over granulopoiesis. *Blood* 119:4408–18. DOI: 10.1182/blood-2011-12-397091.

Györy I, Boller S, Nechanitzky R, Mandel E, Pott S, Liu E, Grosschedl R. 2012. Transcription factor Ebf1 regulates differentiation stage-specific signaling, proliferation, and survival of B cells. *Genes & development* 26:668–82. DOI: 10.1101/gad.187328.112.

Hart MR, Anderson DJ, Porter CC, Neff T, Levin M, Horwitz MS. 2018. Activating PAX gene family paralogs to complement PAX5 leukemia driver mutations. *PLOS Genetics* 14:e1007642. DOI: 10.1371/journal.pgen.1007642.

Herglotz J, Unrau L, Hauschildt F, Fischer M, Kriebitzsch N, Alawi M, Indenbirken D, Spohn M, Muller U, Ziegler M, Schuh W, Jack H-M, Stocking C. 2016. Essential control of early B-cell development by Mef2 transcription factors. *Blood* 127:572–581. DOI: 10.1182/blood-2015-04-643270.

Herzog S, Hug E, Meixlsperger S, Paik J-H, DePinho R a, Reth M, Jumaa H. 2008. SLP-65 regulates immunoglobulin light chain gene recombination through the PI(3)K-PKB-Foxo pathway. *Nature immunology* 9:623–631. DOI: 10.1038/ni.1616.

Hirokawa S, Sato H, Kato I, Kudo A. 2003. EBF-regulating Pax5 transcription is enhanced by STAT5 in the early stage of B cells. *European Journal of Immunology* 33:1824–1829. DOI: 10.1002/eji.200323974.

Hodawadekar S, Park K, Farrar MA, Atchison ML. 2012. A Developmentally Controlled Competitive STAT5–PU.1 DNA Binding Mechanism Regulates Activity of the Ig κE3′ Enhancer. *The Journal of Immunology* 188:2276–2284. DOI: 10.4049/JIMMUNOL.1102239.

Holmes ML, Carotta S, Corcoran LM, Nutt SL. 2006. Repression of Flt3 by Pax5 is crucial for B-cell lineage commitment. *Genes & development* 20:933–8. DOI: 10.1101/gad.1396206.

Huang G, Zhang P, Hirai H, Elf S, Yan X, Chen Z, Koschmieder S, Okuno Y, Dayaram T, Growney JD, Shivdasani RA, Gilliland DG, Speck NA, Nimer SD, Tenen DG. 2008. PU.1 is a major downstream target of AML1 (RUNX1) in adult mouse hematopoiesis. *Nature Genetics* 40:51–60. DOI: 10.1038/ng.2007.7.

Johnson K, Hashimshony T, Sawai CM, Pongubala JM, Skok JA, Aifantis I, Singh H. 2008. Regulation of Immunoglobulin Light-Chain Recombination by the Transcription Factor IRF-4 and the Attenuation of Interleukin-7 Signaling. *Immunity* 28:335–345. DOI: 10.1016/j.immuni.2007.12.019.

Joshi I, Yoshida T, Jena N, Qi X, Zhang J, Van Etten RA, Georgopoulos K. 2014. Loss of Ikaros DNA-binding function confers integrin-dependent survival on pre-B cells and progression to acute lymphoblastic leukemia. *Nature immunology* 15:294–304. DOI: 10.1038/ni.2821.

Karamitros D, Stoilova B, Aboukhalil Z, Hamey F, Reinisch A, Samitsch M, et al. 2018. Single-cell analysis reveals the continuum of human lympho-myeloid progenitor cells. *Nature immunology* 19:85–97. DOI: 10.1038/s41590-017-0001-2.

Katerndahl CDS, Heltemes-Harris LM, Willette MJL, Henzler CM, Frietze S, Yang R, et al. 2017. Antagonism of B cell enhancer networks by STAT5 drives leukemia and poor patient survival. *Nature immunology* 18:694–704. DOI: 10.1038/ni.3716.

Kersseboom R, Middendorp S, Dingjan GM, Dahlenborg K, Reth M, Jumaa H, Hendriks RW. 2003. Bruton’s tyrosine kinase cooperates with the B cell linker protein SLP-65 as a tumor suppressor in Pre-B cells. *The Journal of experimental medicine* 198:91–8. DOI: 10.1084/jem.20030615.

Kikuchi K, Kasai H, Watanabe A, Lai AY, Kondo M. 2008. IL-7 specifies B cell fate at the common lymphoid progenitor to pre-proB transition stage by maintaining early B cell factor expression. *Journal of immunology (Baltimore, Md. : 1950)* 181:383–92.

Kwon K, Hutter C, Sun Q, Bilic I, Cobaleda C, Malin S, Busslinger M. 2008. Instructive Role of the Transcription Factor E2A in Early B Lymphopoiesis and Germinal Center B Cell Development. *Immunity* 28:751–762. DOI: 10.1016/J.IMMUNI.2008.04.014.

Laslo P, Spooner CJ, Warmflash A, Lancki DW, Lee H-J, Sciammas R, Gantner BN, Dinner AR, Singh H. 2006. Multilineage Transcriptional Priming and Determination of Alternate Hematopoietic Cell Fates. *Cell* 126:755–766. DOI: 10.1016/j.cell.2006.06.052.

Lévesque J, Hendy J, Takamatsu Y, Simmons PJ, Bendall LJ. 2003. Disruption of the CXCR4 / CXCL12 chemotactic interaction during hematopoietic stem cell mobilization induced by GCSF or cyclophosphamide. *The Journal of clinical investigation* 111:187–196. DOI: 10.1172/JCI200315994.Introduction.

Li L-X, Goetz CA, Katerndahl CDS, Sakaguchi N, Farrar MA. 2010. A Flt3- and Ras-dependent pathway primes B cell development by inducing a state of IL-7 responsiveness. *Journal of immunology (Baltimore, Md. : 1950)* 184:1728–36. DOI: 10.4049/jimmunol.0903023.

Li X, Xu Z, Du W, Zhang Z, Wei Y, Wang H, Zhu Z, Qin L, Wang L, Niu Q, Zhao X, Girard L, Gong Y, Ma Z, Sun B, Yao Z, Minna JD, Terada LS, Liu Z. 2014. Aiolos Promotes Anchorage Independence by Silencing p66 Shc Transcription in Cancer Cells. *Cancer Cell* 25:575–589. DOI: 10.1016/j.ccr.2014.03.020.

Lidonnici MR, Audia A, Soliera AR, Prisco M, Ferrari-Amorotti G, Waldron T, Donato N, Zhang Y, Martinez R V., Holyoake TL, Calabretta B. 2010. Expression of the Transcriptional Repressor Gfi-1 Is Regulated by C/EBP  and Is Involved in Its Proliferation and Colony Formation-Inhibitory Effects in p210BCR/ABL-Expressing Cells. *Cancer Research* 70:7949–7959. DOI: 10.1158/0008-5472.CAN-10-1667.

Lu D, Nakagawa R, Lazzaro S, Staudacher P, Abreu-Goodger C, Henley T, et al. 2014. The miR-155–PU.1 axis acts on Pax5 to enable efficient terminal B cell differentiation. *The Journal of Experimental Medicine* 211:2183. DOI: 10.1084/JEM.20140338.

Ma S, Pathak S, Trinh L, Lu R. 2008. Interferon regulatory factors 4 and 8 induce the expression of Ikaros and Aiolos to down-regulate pre-B-cell receptor and promote cell-cycle withdrawal in pre-B-cell development. *Blood* 111:1396–1403. DOI: 10.1182/blood-2007-08-110106.

Ma S, Turetsky A, Trinh L, Lu R. 2006. IFN regulatory factor 4 and 8 promote Ig light chain kappa locus activation in pre-B cell development. *Journal of immunology (Baltimore, Md. : 1950)* 177:7898–904.

Mansson R, Welinder E, Åhsberg J, Lin YC, Benner C, Glass CK, Lucas JS, Sigvardsson M, Murre C. 2012. Positive intergenic feedback circuitry, involving EBF1 and FOXO1, orchestrates B-cell fate. *Proceedings of the National Academy of Sciences of the United States of America* 109:21028–33. DOI: 10.1073/pnas.1211427109.

Marteijn JAF, van der Meer LT, Van Emst L, de Witte T, Jansen JH, van der Reijden BA. 2007. Diminished proteasomal degradation results in accumulation of Gfi1 protein in monocytes. *Blood* 109:100–108. DOI: 10.1182/blood-2006-02-003590.

Meek K, Xu Y, Bailie C, Yu K, Neal JA. 2016. The ATM Kinase Restrains Joining of Both VDJ Signal and Coding Ends. *Journal of immunology (Baltimore, Md. : 1950)* 197:3165–3174. DOI: 10.4049/jimmunol.1600597.

Mendoza L, Méndez A. 2015. A dynamical model of the regulatory network controlling lymphopoiesis. *Biosystems* 137:26–33. DOI: 10.1016/j.biosystems.2015.09.004.

Mendoza L, Xenarios I. 2006. A method for the generation of standardized qualitative dynamical systems of regulatory networks. *Theoretical biology & medical modelling* 3:13. DOI: 10.1186/1742-4682-3-13.

Min IM, Pietramaggiori G, Kim FS, Passegué E, Stevenson KE, Wagers AJ. 2008. The Transcription Factor EGR1 Controls Both the Proliferation and Localization of Hematopoietic Stem Cells. *Cell Stem Cell* 2:380–391. DOI: 10.1016/J.STEM.2008.01.015.

Mullighan CG, Miller CB, Radtke I, Phillips L a, Dalton J, Ma J, White D, Hughes TP, Le Beau MM, Pui C-H, Relling M V, Shurtleff S a, Downing JR. 2008. BCR-ABL1 lymphoblastic leukaemia is characterized by the deletion of Ikaros. *Nature* 453:110–4. DOI: 10.1038/nature06866.

Müssel C, Hopfensitz M, Kestler H a. 2010. BoolNet-an R package for generation, reconstruction and analysis of Boolean networks. *Bioinformatics* 26:1378–1380. DOI: 10.1093/bioinformatics/btq124.

Nakayama J, Yamamoto M, Hayashi K, Satoh H, Bundo K, Kubo M, Goitsuka R, Farrar MA, Kitamura D. 2008. BLNK suppresses pre-B-cell leukemogenesis through inhibition of JAK3. *Blood* 113:1483–1492. DOI: 10.1182/blood-2008-07-166355.

Nichogiannopoulou A, Trevisan M, Neben S, Friedrich C, Georgopoulos K. 1999. Defects in hemopoietic stem cell activity in Ikaros mutant mice. *The Journal of experimental medicine* 190:1201–14. DOI: 10.1084/JEM.190.9.1201.

Nodland SE, Berkowska M a, Bajer A a, Shah N, de Ridder D, van Dongen JJM, LeBien TW, van Zelm MC. 2011. IL-7R expression and IL-7 signaling confer a distinct phenotype on developing human B-lineage cells. *Blood* 118:2116–27. DOI: 10.1182/blood-2010-08-302513.

O’Riordan M, Grosschedl R. 1999. Coordinate Regulation of B Cell Differentiation by the Transcription Factors EBF and E2A. *Immunity* 11:21–31. DOI: 10.1016/S1074-7613(00)80078-3.

Ochiai K, Maienschein-Cline M, Mandal M, Triggs JR, Bertolino E, Sciammas R, Dinner AR, Clark MR, Singh H. 2012. A self-reinforcing regulatory network triggered by limiting IL-7 activates pre-BCR signaling and differentiation. *Nature Immunology* 13:300–307. DOI: 10.1038/ni.2210.

Ochodnicka-Mackovicova K, Bahjat M, Bloedjes TA, Maas C, Bruin AM De, Bende RJ, Noesel CJM Van, Guikema JEJ. 2015. NF-kB and AKT signaling prevent DNA damage in transformed pre-B cells by suppressing RAG1 / 2 expression and activity. *Blood* 126:1324–1335. DOI: 10.1182/blood-2015-01-621623.K.O.-M.

Ochodnicka-Mackovicova K, Bahjat M, Maas C, van der Veen A, Bloedjes TA, de Bruin AM, et al. 2016. The DNA Damage Response Regulates RAG1/2 Expression in Pre–B Cells through ATM-FOXO1 Signaling. *The Journal of Immunology* 197:2918–2929. DOI: 10.4049/jimmunol.1501989.

Papaemmanuil E, Rapado I, Li Y, Potter NE, Wedge DC, Tubio J, et al. 2014. RAG-mediated recombination is the predominant driver of oncogenic rearrangement in ETV6-RUNX1 acute lymphoblastic leukemia. *Nature genetics* 46:116–25. DOI: 10.1038/ng.2874.

Pappu R, Cheng AM, Li B, Gong Q, Chiu C, Griffin N, White M, Sleckman BP, Chan AC. 1999. Requirement for B cell linker protein (BLNK) in B cell development. *Science (New York, N.Y.)* 286:1949–54.

Park S-Y, Wolfram P, Canty K, Harley B, Nombela-Arrieta C, Pivarnik G, Manis J, Beggs HE, Silberstein LE. 2013. Focal adhesion kinase regulates the localization and retention of pro-B cells in bone marrow microenvironments. *The Journal of Immunology* 190:1094–102. DOI: 10.4049/jimmunol.1202639.

Peled A, Kollet O, Ponomaryov T, Petit I, Franitza S, Grabovsky V, et al. 2000. The chemokine SDF-1 activates the integrins LFA-1, VLA-4, and VLA-5 on immature human CD34(+) cells: role in transendothelial/stromal migration and engraftment of NOD/SCID mice. *Blood* 95:3289–96.

Pérez-Saldivar ML, Fajardo-Gutiérrez A, Bernáldez-Ríos R, Martínez-Avalos A, Medina-Sanson A, Espinosa-Hernández L, et al. 2011. Childhood acute leukemias are frequent in Mexico City: descriptive epidemiology. *BMC cancer* 11:355. DOI: 10.1186/1471-2407-11-355.

Perfetti V, Vignarelli MC, Palladini G, Navazza V, Giachino C, Merlini G. 2004. Insights into the regulation of immunoglobulin light chain gene rearrangements via analysis of the kappa light chain locus in lambda myeloma. *Immunology* 112:420–7. DOI: 10.1046/j.1365-2567.2004.01902.x.

Pongubala JMR, Northrup DL, Lancki DW, Medina KL, Treiber T, Bertolino E, et al. 2008. Transcription factor EBF restricts alternative lineage options and promotes B cell fate commitment independently of Pax5. *Nature Immunology* 9:203–215. DOI: 10.1038/ni1555.

de Pooter RF, Kee BL. 2010. E proteins and the regulation of early lymphocyte development. *Immunological reviews* 238:93–109. DOI: 10.1111/j.1600-065X.2010.00957.x.

Rao KN, Smuda C, Gregory GD, Min B, Brown MA. 2013. Ikaros limits basophil development by suppressing C/EBP-  expression. *Blood* 122:2572–2581. DOI: 10.1182/blood-2013-04-494625.

Roessler S, Gyory I, Imhof S, Spivakov M, Williams RR, Busslinger M, Fisher AG, Grosschedl R. 2007. Distinct Promoters Mediate the Regulation of Ebf1 Gene Expression by Interleukin-7 and Pax5. *Molecular and Cellular Biology* 27:579–594. DOI: 10.1128/MCB.01192-06.

Rogers JH, Owens KS, Kurkewich J, Klopfenstein N, Iyer SR, Simon MC, Dahl R. 2016. E2A Antagonizes PU.1 Activity through Inhibition of DNA Binding. *BioMed Research International* 2016:1–11. DOI: 10.1155/2016/3983686.

Schebesta M, Pfeffer PL, Busslinger M. 2002. Control of pre-BCR signaling by Pax5-dependent activation of the BLNK gene. *Immunity* 17:473–85. DOI: 10.1016/S1074-7613(02)00418-1.

Seo W, Ikawa T, Kawamoto H, Taniuchi I. 2012. Runx1-Cbfβ facilitates early B lymphocyte development by regulating expression of Ebf1. *The Journal of experimental medicine* 209:1255–62. DOI: 10.1084/jem.20112745.

Simonetti G, Carette A, Silva K, Wang H, De Silva NS, Heise N, Siebel CW, Shlomchik MJ, Klein U. 2013. IRF4 controls the positioning of mature B cells in the lymphoid microenvironments by regulating NOTCH2 expression and activity. *Journal of Experimental Medicine* 210.

Singh H, Pongubala JMR, Medina KL. 2007. Gene regulatory networks that orchestrate the development of B lymphocyte precursors. *Advances in experimental medicine and biology* 596:57–62. DOI: 10.1007/0-387-46530-8_5.

Spooner CJ, Cheng JX, Pujadas E, Laslo P, Singh H. 2009. A Recurrent Network Involving the Transcription Factors PU.1 and Gfi1 Orchestrates Innate and Adaptive Immune Cell Fates. *Immunity* 31:576–586. DOI: 10.1016/j.immuni.2009.07.011.

Stehling-Sun S, Dade J, Nutt SL, DeKoter RP, Camargo FD. 2009. Regulation of lymphoid versus myeloid fate “choice” by the transcription factor Mef2c. *Nature Immunology* 10:289–296. DOI: 10.1038/ni.1694.

Sugiyama T, Kohara H, Noda M, Nagasawa T. 2006. Maintenance of the hematopoietic stem cell pool by CXCL12-CXCR4 chemokine signaling in bone marrow stromal cell niches. *Immunity* 25:977–88. DOI: 10.1016/j.immuni.2006.10.016.

Tabe Y, Jin L, Tsutsumi-Ishii Y, Xu Y, McQueen T, Priebe W, Mills GB, Ohsaka A, Nagaoka I, Andreeff M, Konopleva M. 2007. Activation of integrin-linked kinase is a critical prosurvival pathway induced in leukemic cells by bone marrow-derived stromal cells. *Cancer Research* 67:684–694. DOI: 10.1158/0008-5472.CAN-06-3166.

Tarnowski M, Kucia M, Ratajczak MZ. 2009. Isolation and Functional Analysis of CXCR7 Promoter - a Novel Receptor for Stromal Derived Factor-1 (SDF-1): Different Regulation of Expression in Human Hematopoietic Cells Versus Pediatric Sarcomas. *Blood* 114.

Tarnowski M, Liu R, Wysoczynski M, Ratajczak J, Kucia M, Ratajczak MZ. 2010. CXCR7: A new SDF-1-binding receptor in contrast to normal CD34+ progenitors is functional and is expressed at higher level in human malignant hematopoietic cells. *European Journal of Haematology* 85:472–483. DOI: 10.1111/j.1600-0609.2010.01531.x.

Terashima A, Okamoto K, Nakashima T, Akira S, Ikuta K, Takayanagi H. 2016. Sepsis-Induced Osteoblast Ablation Causes Immunodeficiency. *Immunity* 44:1434–1443. DOI: 10.1016/j.immuni.2016.05.012.

Thompson EC, Cobb BS, Sabbattini P, Meixlsperger S, Parelho V, Liberg D, Taylor B, Dillon N, Georgopoulos K, Jumaa H, Smale ST, Fisher AG, Merkenschlager M. 2007. Ikaros DNA-Binding Proteins as Integral Components of B Cell Developmental-Stage-Specific Regulatory Circuits. *Immunity* 26:335–344. DOI: 10.1016/j.immuni.2007.02.010.

Tokoyoda K, Egawa T, Sugiyama T, Choi B-I, Nagasawa T. 2004. Cellular niches controlling B lymphocyte behavior within bone marrow during development. *Immunity* 20:707–18. DOI: 10.1016/j.immuni.2004.05.001.

Torossian F, Anginot A, Chabanon A, Clay D, Guerton B, Desterke C, et al. 2014. CXCR7 participates in CXCL12-induced CD34+ cell cycling through b-arrestin–dependent Akt activation. 123:191–202. DOI: 10.1182/blood-2013-05-500496.

Übelhart R, Bach MP, Eschbach C, Wossning T, Reth M, Jumaa H. 2010. N-linked glycosylation selectively regulates autonomous precursor BCR function. *Nature Immunology* 11:759–765. DOI: 10.1038/ni.1903.

Uto-Konomi A, McKibben B, Wirtz J, Sato Y, Takano A, Nanki T, Suzuki S. 2013. CXCR7 agonists inhibit the function of CXCL12 by down-regulation of CXCR4. *Biochemical and Biophysical Research Communications* 431:772–776. DOI: 10.1016/j.bbrc.2013.01.032.

Villarreal C, Padilla-Longoria P, Alvarez-Buylla ER. 2012. General Theory of Genotype to Phenotype Mapping: Derivation of Epigenetic Landscapes from N-Node Complex Gene Regulatory Networks. *Physical Review Letters* 109:118102. DOI: 10.1103/PhysRevLett.109.118102.

Wang H, Guan X, Tu Y, Zheng S, Long J, Li S, Qi C, Xie X, Zhang H, Zhang Y. 2015. MicroRNA-29b attenuates non-small cell lung cancer metastasis by targeting matrix metalloproteinase 2 and PTEN. *Journal of Experimental & Clinical Cancer Research* 34:59. DOI: 10.1186/s13046-015-0169-y.

Welinder E, Mansson R, Mercer EM, Bryder D, Sigvardsson M, Murre C. 2011. The transcription factors E2A and HEB act in concert to induce the expression of FOXO1 in the common lymphoid progenitor. *Proceedings of the National Academy of Sciences of the United States of America* 108:17402–7. DOI: 10.1073/pnas.1111766108.

Welner RS, Pelayo R, Kincade PW. 2008. Evolving views on the genealogy of B cells. *Nature reviews. Immunology* 8:95–106. DOI: 10.1038/nri2234.

Yücel R, Kosan C, Heyd F, Möröy T. 2004. Gfi1: Green fluorescent protein knock-in mutant reveals differential expression and autoregulation of the growth factor independence 1 (Gfi1) gene during lymphocyte development. *The Journal of biological chemistry* 279:40906–17. DOI: 10.1074/jbc.M400808200.

Zandi S, Mansson R, Tsapogas P, Zetterblad J, Bryder D, Sigvardsson M. 2008. EBF1 Is Essential for B-Lineage Priming and Establishment of a Transcription Factor Network in Common Lymphoid Progenitors. *The Journal of Immunology* 181:3364–3372. DOI: 10.4049/jimmunol.181.5.3364.

Zarnegar MA, Rothenberg E V. 2012. Ikaros represses and activates PU.1 cell-type-specifically through the multifunctional Sfpi1 URE and a myeloid specific enhancer. *Oncogene* 31:4647–54. DOI: 10.1038/onc.2011.597.

Zehentmeier S, Pereira JP. 2019. Cell circuits and niches controlling B cell development. *Immunological Reviews* 289:142–157. DOI: 10.1111/imr.12749.

Zhou N, Gutierrez-Uzquiza A, Zheng XY, Chang R, Vogl DT, Garfall AL, Bernabei L, Saraf A, Florens L, Washburn MP, Illendula A, Bushweller JH, Busino L. 2019. RUNX proteins desensitize multiple myeloma to lenalidomide via protecting IKZFs from degradation. *Leukemia*:1. DOI: 10.1038/s41375-019-0403-2.
