# Supplementary material for: Dynamical modeling predicts an inflammation-inducible CXCR7+ B cell precursor with potential implications in lymphoid blockage pathologies
Source: PeerJ. 2020 Sep 29;8:e9902. doi: 10.7717/peerj.9902 (PMC7531334; doi:10.7717/peerj.9902)
Supplement: Table S2 — The logical rules of the reduced components were replaced in their target nodes. The reduced nodes and replaced logical rules are identified by gray color. Boolean operators are represented with the symbols & (AND), | (OR) and ! (NOT). [file peerj-08-9902-s002.doc]

**Supplementary Table 2.** **Logical rules used for computational simulation of the eBCRN.** The logical rules of the reduced components were replaced in their target nodes. The reduced nodes and replaced logical rules are identified by gray color. Boolean operators are represented with the symbols & (AND), | (OR) and ! (NOT).

| **Node** | **Boolean rule** |
| --- | --- |
| Flt3L | Flt3L |
| Il7 | Il7 |
| BCR | Spi1 & Tcf3 & Irf4 & !STAT5 & (Rag | BCR) |
|  | Spi1 & Tcf3 & Irf4 & !STAT5 & ((Ebf1 & Foxo1 & !(SLP65 \| NFkB)) \| BCR) |
| VCAM1_VLA4 | (Spi1 | Spi1_2) & !(Ikzf1 & Ikzf3) |
|  | (Spi1 \| Spi1_2) & !(Ikzf1 & Irf4 & !STAT5) |
| preBCR | Ebf1 & !Ikzf3 & (Rag|preBCR) |
|  | Ebf1 & !(Ikzf1 & Irf4 & !STAT5) & ((Ebf1 & Foxo1 & !(SLP65 \| NFkB))\|preBCR) |
| Csf1r | Spi1_2 & !Pax5 |
| Cxcr4 | Tcf3 & (!Gfi1 | Flt3_a | Irf4) & !Cxcr7 |
|  | Tcf3 & (!Gfi1 \| (Flt3 & Flt3L) \| Irf4) & !Cxcr7 |
| Cxcr7 | NFkB & !Irf4 |
| Flt3 | Ikzf1 & Spi1 & !Pax5 |
| Flt3_a | Flt3 & Flt3L |
| Il7r | Spi1 & Foxo1 & (Flt3_a | (STAT5 & Ebf1)) & !(Cebpa | Irf4) |
|  | Spi1 & Foxo1 & ((Flt3 & Flt3L) \| (STAT5 & Ebf1)) & !(Cebpa \| Irf4) |
| Il7r_a | Il7r & Il7 |
| STAT5 | Il7r_a & VCAM1_VLA4 & !SLP65 |
|  | Il7r & Il7 & VCAM1_VLA4 & !SLP65 |
| PI3KIA | (preBCR | (VCAM1_VLA4 & Cxcr4) | Cxcr7) & !SLP65 & (!Cebpa | STAT5) |
| NFkB | SLP65 | Rag |
|  | SLP65 \| (Ebf1 & Foxo1 & !(SLP65 \| NFkB)) |
| SLP65 | preBCR & (Foxo1 | Pax5) |
| Cebpa | (Spi1 | Runx1) & (!(Ebf1 | Foxo1 | Ikzf1) | (Runx1 & Spi1_2)) |
| Ebf1 | Tcf3 & Runx1 & ((STAT5 & Foxo1) | (Spi1 & Ebf1 & Pax5)) & !Cebpa |
| Egr1 | (Spi1 & !Gfi1) | Spi1_2 | Pax5 |
| Foxo1 | (Tcf3 | Ebf1) & (!PI3KIA | !NFkB | SLP65) & !Cebpa |
| Gfi1 | ((Ikzf1 | Cebpa) & !(Egr1 | Gfi1) | Ebf1) |
| Ikzf1 | (Runx1 & Spi1 & !Cebpa) | Irf4 |
| Ikzf3 | Ikzf1 & Irf4 & !STAT5 |
| Irf4 | (NFkB | Ebf1) & !((Flt3 & Flt3L) | STAT5) |
| Pax5 | Spi1 & (STAT5 | (Ebf1 & Foxo1 & Irf4)) & !Cebpa |
| Rag | Ebf1 & Foxo1 & !(SLP65 | NFkB) |
| Spi1 | Runx1 & ((Gfi1 | Tcf3) | (Spi1 & Ikzf1)) & !Cebpa |
| Spi1_2 | Runx1 & (!(Gfi1 | Tcf3) | (Spi1_2 & !Ikzf1)) |
| Tcf3 | (Ikzf1 | Spi1 | Pax5) & (Tcf3 | Ebf1 | Gfi1) |
| Runx1 | (Spi1 | Spi1_2) & (Runx1 | Ikzf1) |
