# Supplementary material for: Dynamical modeling predicts an inflammation-inducible CXCR7+ B cell precursor with potential implications in lymphoid blockage pathologies
Source: PeerJ. 2020 Sep 29;8:e9902. doi: 10.7717/peerj.9902 (PMC7531334; doi:10.7717/peerj.9902)
Supplement: Table S3 — KO, Knock out; OA, overactivation. [file peerj-08-9902-s003.doc]

**Supplementary Table 3. Extended results and analyses from the continuous simulations of all possible mutant networks.** KO, Knock out; OA, overactivation.

|  | **Node** | **Mut** | **Model interpretation** | **Observation** | **References** |
| --- | --- | --- | --- | --- | --- |
|  | Csf1r | KO | No effect in the transcriptional core during B cell differentiation | Output | |
|  | BCR | KO |  | Output | |
|  | Flt3L | OA |  | Input | |
|  | IL7 | OA |  | Input | |
|  | BCR | OA |  | Output | |
|  | Csf1r | OA |  | Output | |
|  | Ikzf1 | OA |  | No effect, as they are active since the LMPP stage and maintain their activation through all early B cell differentiation stages recovered by the wild-type model. | |
|  | Spi1 | OA |  |  |  |
|  | Runx1 | OA |  |  |  |
|  | Cxcr7 | KO |  | No effect, in concordance with its low expression in normal lymphoid cell lines and hematopoietic progenitors. | (Tarnowski et al., 2010) |
|  | Egr1 | KO |  | Egr1 do not have an evident role in the lymphopoietic transcription factor network, at least until cells migrate to the spleen, where B cells upregulate their expression. | (Collombet et al., 2017) |
|  | VCAM1_VLA4 | OA |  | VCAM-1/VLA-4 involvement in lymphoid differentiation, is related to migration between niches and not directly to an effect in the transcriptional network. | (Peled et al., 2000) |
|  | PI3KIA | OA | Perturbation at the pro-B/pre-B cell stage without affecting the differentiation process | PI3KIA overactivation affects FOXO1 at the pre-B stage when NFkB also, a FOXO1 repressor, is activated downstream the pre-BCR. The inhibition of PTEN, a PI3K regulator, revealed only poor reduction in cell numbers of the developmental stages following pro-B cells. | (Powers et al., 2012) |
|  | PI3KIA | KO | Determinant during pre-B cell stage, but not in the main core Regulating cellular transition to immature B-cell | In mice deficient for the regulatory or the catalytic subunit of PI3K Class I, B cell development was slightly blocked at the pre-B cell stage. | (Fruman et al., 1999; Clayton et al., 2002; Jou et al., 2002) |
|  | Cxcr4 | KO |  | Pro-B and pre-B cell numbers were severely reduced in the BM of mutant mice with a disrupted expression of SDF-1/CXCL12 or its receptor CXCR4. | (Nagasawa et al., 1996; Ma et al., 1998) |
|  | Cxcr4 | OA |  | Overexpression of CXCR4 in hematopoietic progenitor cells alter their homming and reconstitution potential, having a role on migration and proliferation. | (Kahn et al., 2004) |
|  | Cebpa | KO |  | Absence of C/EBPα prevents formation of GMPs and myeloid colony-forming units, in addition it has been reported that LMPPs keep some capacity to generate granulocytic, monocyte/macrophage and granulocyte-macrophage colonies. When Cebpa is inhibited, the transition towards Immature B cell is reduced and in consequence, some intermediate stages are outdated. | (Heath et al., 2004; Karamitros et al., 2018) |
|  | Spi1_2 | KO |  | Similar to Cebpa, Spi1_2 mutation in the model inhibits, since the first time-steps the option of activating myeloid lineage elements, accelerating the establishment of the nodes determining the lymhphoid lineage. | Inferred explanation, no specific reference. |
|  | Cxcr7 | OA | No clear effect | The effect of Cxcr7 on PI3K and Cxcr4 activation is not sufficient to affect B cell differentiation, but generate transient perturbations in two particular stages: prepro-B and pre-B. | (Melo et al., 2014) |
|  | Irf4 | KO | B cell arrest at late pro-B/pre-B stage | B-cell development of IRF-4,8-/- double mutant mice, is arrested at the pre-B stage. IRF-4 and IRF-8 have redundant activities, but IRF-8 was not included in the current model. | (Lu et al., 2003) |
|  | Flt3 | OA |  | Enforced expression of FLT3 in mice HSC affects B-cell precursors frequency, CLP transitioning to B cell progenitors, but not committed B cells. | (Holmes et al., 2006) |
|  | SLP65 | KO |  | Inadequate signaling of pre-BCR, as observed with BLNK gene mutations, induces a block in B-cell development at the pro–B-cell to pre–B-cell transition. | (Minegishi et al., 1999) |
|  | preBCR | KO |  |  |  |
|  | Pax5 | KO |  | Pax-5-/- mice do not commit to the immature B stage and get arrested at the pro-B stage. | (Urbánek et al., 1994; Cobaleda et al., 2007)⁠ |
|  | STAT5 | KO | Blockage CLP or early pre-pro-B stage | STAT5 knock-outs lead to hematopoietic defects with particular deficiency in pre-proB or pro-B development. | (Teglund et al., 1998; Goetz et al., 2004; Yao et al., 2006) |
|  | Il7r | KO |  | Adult mice expressing mutated IL-7, or any of the two chains comprising IL-7R, show a significant arrest at the pre–pro-B cell stage or even early, at the CLP stage. | (Miller et al., 2002; Kikuchi et al., 2005) |
|  | Ebf1 | KO |  | EBF deficient mice lack immunoglobulin-expressing B-cells, but contain progenitor cells expressing IL-7 receptor transcripts | (Lin & Grosschedl, 1995) |
|  | NFkB | KO |  | Loss of NF-kB pathway members leads to an accumulation of HSCs and a decrease of myeloid and lymphoid progenitors. Additionally, the inhibition of NF-kB activity on pro-B cells, inhibits light chain rearrangements for pre-BCR. | (Cadera et al., 2009; Stein & Baldwin, 2013) |
|  | Foxo1 | KO |  | Deletion of Foxo 1,3 and 4 in HSCs leads to a severe block in the generation of CLPs. While, Foxo1 conditional mutation in early B-cell progenitors derives in a reduction of immature and circulating B-cells, and an increase in the percentage of BM pro-B cells. | (Tothova et al., 2007; Dengler et al., 2008) |
|  | VCAM1_VLA4 | KO |  | Mice chimeric for the expression of α4, a subunit of VLA-4 integrin, showed a significant reduction on pre-B cells and mature B cells, suggesting a severe failure in B cell development before pre-B stage. | (Arroyo et al., 1996) |
|  | Flt3 | KO |  | FLT3 conditional KO significantly decreases the numbers of LMPPs and ETPs in adult mice, but not CLPs and B cell progenitors. However, Flt3-deleted LMPPs or CLPs, showed a disadvantage in producing B-cell progenitors compared to non-deleted progenitors. | (Zriwil et al., 2018) |
|  | preBCR | OA |  | There is no biological interpretation, as there is no report of pre-BCR expression and activation at an earlier stage. However, pre-BCR constitutive activiation at the pro-B stage, derives in enhanced proliferation without progressing to pre-B cell. | (Schebesta, Pfeffer & Busslinger, 2002) |
|  | Irf4 | OA |  | Irf4 OA, blocks simulation before CLP stage because because it inhibits IL7r activation. | (Johnson et al., 2008; Ma et al., 2008) |
|  | Il7 | KO |  | Adult mice expressing mutated IL-7, or any of the two chains comprising IL-7R, show a significant arrest at the pre–pro-B cell stage or even early, at the CLP stage. | (Miller et al., 2002; Kikuchi et al., 2005) |
|  | Flt3L | KO |  | Flt3 deficient mice showed a reduction in leukocyte cellularity and a severe ablation of dendritic and NK cells, suggesting that it affects at a progenitor stage. | (McKenna et al., 2000) |
|  | SLP65 | OA |  | There is no biological interpretation, as there is no report of pre-BCR expression and activation at an earlier stage. However, pre-BCR constitutive activiation at the pro-B stage, derives in enhanced proliferation without progressing to pre-B cell. | (Schebesta, Pfeffer & Busslinger, 2002) |
|  | NFkB | OA |  | Inflammation, probably mediated by NF-kB responses, induce lymphocyte mobilization to extramedullar niches and enhances granulopoiesis in the BM. However its induction in the model blocks differentiation at the CLP due to the negative regulation of Rag and early IRF4 activation. | (Ueda et al., 2004; Enciso, Mendoza & Pelayo, 2015) |
|  | Tcf3 | KO |  | E2A deletion completely inhibits immunoglobulin gene recombination for B cell production but has little effect on T lineage precursors, suggesting that it is dispensable for ELP production but blocks CLP progression to pro-B cells. | (Bain et al., 1997; Borghesi et al., 2005) |
|  | Spi1_2 | OA | Complete B cell blockage at LMPP | Overexpression of PU. 1 in normal hematopoietic progenitors blocks B cell development | (DeKoter, Lee & Singh, 2002) |
|  | Gfi1 | KO |  | Gfi1 deficiency impairs lymphopoiesis in MPPs and LMPPs showing to be critical to sustain B-cell commitment. | (Spooner et al., 2009; Fraszczak et al., 2016) |
|  | Egr1 | OA |  | No reports of Egr1 OA effects on early B-cell differentiation were found. However, its increased expression has been associated to maintance of LT-HSC, and development of leukemia. | (Tian et al., 2016) |
|  | Cebpa | OA |  | C/EBPα levels increase as long-term stem cells progress to granulocyte–monocyte progenitors (GMP). Over-expression of WT C/EBPα in cordon blood-derived Lin- cells, led to a significant reduction in the total number of CFUs. | (Quintana-Bustamante et al., 2012) |
|  | Runx1 | KO |  | RUNX1 is required for the maintenance of platelets and lymphocytes, probably with a greater participation during development than in adult hematopoiesis. | (Ichikawa et al., 2004) |
|  | Spi1 | KO |  | PU.1 -/- hematopoietic multipotential progenitors produce defects in lymphoid differentiation at a progenitr stage, in addition to the effects caused by the loss of IL-7R expression. | (DeKoter, Lee & Singh, 2002) |
|  | Ikzf1 | KO |  | Null Ikaros mice lack B lymphocytes and their earliest progrenitors | (Wang et al., 1996) |
|  | Foxo1 | OA |  | Forced activation of FOXO1 lower the proliferation rate of ALL cells. | (Köhrer et al., 2016) |
|  | Il7r | OA |  | Gain-of-function mutations in acute lymphoblastic leukemias has been associated to an enhanced growth of progenitor lymphoid cells. | (Shochat et al., 2011) |
|  | Pax5 | OA |  | Pax5 overactivation produces the phenotype reported experimentally for Flt3 KO, where LMPP compartment is affected. Eventhoguh, FLT3 KO in our model didn’t showed this behavior. | (Holmes et al., 2006; Zriwil et al., 2018) |
|  | STAT5 | OA |  | Induced overexpression of STAT5 induces and erythropoiesis through the induction of GATA-1, a transcription factor that participates in a negative feedback loop with the lymphoid factor Ikaros. | (Wierenga, Vellenga & Schuringa, 2010; Malinge et al., 2013) |
|  | Tcf3 | OA | Perturbation during lymphoid commitment without differentiation blockage. | Overexpression of E47, one of the proteins encoded by Tcf3 gene, derives in the induction of cellular proliferation. | (Zhao et al., 2001; Schwartz et al., 2006) |
|  | Gfi1 | OA |  | A role of Gfi1 overactivation in acute lymphoblastic leukemia cells, has been suggested. However its contribution is probably not related to the transcriptional differentiation core but to other related mechanisms involved in lymphopoiesis. | (Purizaca et al., 2013) |
|  | Ebf1 | OA |  | Enforced expression of EBF1 in hematopoietic progenitors, restricts lymphopoiesis to the B cell lineage limiting other lymphoid phenotypes. | (Zhang et al., 2003) |

**References**

Arroyo AG, Yang JT, Rayburn H, Hynes RO. 1996. Differential requirements for alpha4 integrins during fetal and adult hematopoiesis. *Cell* 85:997–1008. DOI: 10.1016/s0092-8674(00)81301-x.

Bain G, Robanus Maandag EC, te Riele HP, Feeney AJ, Sheehy A, Schlissel M, Shinton SA, Hardy RR, Murre C. 1997. Both E12 and E47 allow commitment to the B cell lineage. *Immunity* 6:145–54.

Borghesi L, Aites J, Nelson S, Lefterov P, James P, Gerstein R. 2005. E47 is required for V(D)J recombinase activity in common lymphoid progenitors. *The Journal of experimental medicine* 202:1669–77. DOI: 10.1084/jem.20051190.

Cadera EJ, Wan F, Amin RH, Nolla H, Lenardo MJ, Schlissel MS. 2009. NF-kappaB activity marks cells engaged in receptor editing. *The Journal of experimental medicine* 206:1803–16. DOI: 10.1084/jem.20082815.

Clayton E, Bardi G, Bell SE, Chantry D, Downes CP, Gray A, Humphries LA, Rawlings D, Reynolds H, Vigorito E, Turner M. 2002. A Crucial Role for the p110δ Subunit of Phosphatidylinositol 3-Kinase in B Cell Development and Activation. *The Journal of Experimental Medicine* 196:753–763. DOI: 10.1084/jem.20020805.

Cobaleda C, Schebesta A, Delogu A, Busslinger M. 2007. Pax5: the guardian of B cell identity and function. *Nature Immunology* 8:463–470. DOI: 10.1038/ni1454.

Collombet S, van Oevelen C, Sardina Ortega JL, Abou-Jaoudé W, Di Stefano B, Thomas-Chollier M, Graf T, Thieffry D. 2017. Logical modeling of lymphoid and myeloid cell specification and transdifferentiation. *Proceedings of the National Academy of Sciences of the United States of America* 114:5792–5799. DOI: 10.1073/pnas.1610622114.

DeKoter RP, Lee H-J, Singh H. 2002. PU.1 regulates expression of the interleukin-7 receptor in lymphoid progenitors. *Immunity* 16:297–309. DOI: 10.1016/S1074-7613(02)00269-8.

Dengler HS, Baracho G V, Omori SA, Bruckner S, Arden KC, Castrillon DH, DePinho RA, Rickert RC. 2008. Distinct functions for the transcription factor Foxo1 at various stages of B cell differentiation. *Nature immunology* 9:1388–98. DOI: 10.1038/ni.1667.

Enciso J, Mendoza L, Pelayo R. 2015. Normal vs. Malignant hematopoiesis: the complexity of acute leukemia through systems biology. *Frontiers in Genetics* 6:1–5. DOI: 10.3389/fgene.2015.00290.

Fraszczak J, Helness A, Chen R, Vadnais C, Robert F, Khandanpour C, Möröy T. 2016. Threshold Levels of Gfi1 Maintain E2A Activity for B Cell Commitment via Repression of Id1. *PLOS ONE* 11:e0160344. DOI: 10.1371/journal.pone.0160344.

Fruman DA, Snapper SB, Yballe CM, Davidson L, Yu JY, Alt FW, Cantley LC. 1999. Impaired B Cell Development and Proliferation in Absence of Phosphoinositide 3-Kinase p85. *Science* 283:393–397. DOI: 10.1126/science.283.5400.393.

Goetz CA, Harmon IR, O’Neil JJ, Burchill MA, Farrar MA. 2004. STAT5 activation underlies IL7 receptor-dependent B cell development. *Journal of immunology (Baltimore, Md. : 1950)* 172:4770–8. DOI: 10.4049/jimmunol.172.8.4770.

Heath V, Suh HC, Holman M, Renn K, Gooya JM, Parkin S, Klarmann KD, Ortiz M, Johnson P, Keller J. 2004. C/EBP deficiency results in hyperproliferation of hematopoietic progenitor cells and disrupts macrophage development in vitro and in vivo. *Blood* 104:1639–1647. DOI: 10.1182/blood-2003-11-3963.

Holmes ML, Carotta S, Corcoran LM, Nutt SL. 2006. Repression of Flt3 by Pax5 is crucial for B-cell lineage commitment. *Genes & development* 20:933–8. DOI: 10.1101/gad.1396206.

Ichikawa M, Asai T, Saito T, Yamamoto G, Seo S, Yamazaki I, et al. 2004. AML-1 is required for megakaryocytic maturation and lymphocytic differentiation, but not for maintenance of hematopoietic stem cells in adult hematopoiesis. *Nature Medicine* 10:299–304. DOI: 10.1038/nm997.

Johnson K, Hashimshony T, Sawai CM, Pongubala JM, Skok JA, Aifantis I, Singh H. 2008. Regulation of Immunoglobulin Light-Chain Recombination by the Transcription Factor IRF-4 and the Attenuation of Interleukin-7 Signaling. *Immunity* 28:335–345. DOI: 10.1016/j.immuni.2007.12.019.

Jou S-T, Carpino N, Takahashi Y, Piekorz R, Chao J-R, Carpino N, Wang D, Ihle JN. 2002. Essential, nonredundant role for the phosphoinositide 3-kinase p110delta in signaling by the B-cell receptor complex. *Molecular and cellular biology* 22:8580–91. DOI: 10.1128/mcb.22.24.8580-8591.2002.

Kahn J, Byk T, Jansson-Sjostrand L, Petit I, Shivtiel S, Nagler A, Hardan I, Deutsch V, Gazit Z, Gazit D, Karlsson S, Lapidot T. 2004. Overexpression of CXCR4 on human CD34+ progenitors increases their proliferation, migration, and NOD/SCID repopulation. *Blood* 103:2942–2949. DOI: 10.1182/blood-2003-07-2607.

Karamitros D, Stoilova B, Aboukhalil Z, Hamey F, Reinisch A, Samitsch M, Quek L, Otto G, Repapi E, Doondeea J, Usukhbayar B, Calvo J, Taylor S, Goardon N, Six E, Pflumio F, Porcher C, Majeti R, Göttgens B, Vyas P. 2018. Single-cell analysis reveals the continuum of human lympho-myeloid progenitor cells. *Nature immunology* 19:85–97. DOI: 10.1038/s41590-017-0001-2.

Kikuchi K, Lai AY, Hsu C-L, Kondo M. 2005. IL-7 receptor signaling is necessary for stage transition in adult B cell development through up-regulation of EBF. *The Journal of Experimental Medicine* 201:1197. DOI: 10.1084/JEM.20050158.

Köhrer S, Havranek O, Seyfried F, Hurtz C, Coffey GP, Kim E, et al. 2016. Pre-BCR signaling in precursor B-cell acute lymphoblastic leukemia regulates PI3K/AKT, FOXO1 and MYC, and can be targeted by SYK inhibition. *Leukemia* 30:1246–1254. DOI: 10.1038/leu.2016.9.

Lin H, Grosschedl R. 1995. Failure of B-cell differentiation in mice lacking the transcription factor EBF. *Nature* 376:263–267. DOI: 10.1038/376263a0.

Lu R, Medina KL, Lancki DW, Singh H. 2003. IRF-4,8 orchestrate the pre-B-to-B transition in lymphocyte development. *Genes & development* 17:1703–8. DOI: 10.1101/gad.1104803.

Ma Q, Jones D, Borghesani PR, Segal RA, Nagasawa T, Kishimoto T, Bronson RT, Springer TA. 1998. Impaired B-lymphopoiesis , myelopoiesis , and derailed cerebellar neuron migration in CXCR4- and SDF-1-deficient mice. *Proceedings of the National Academy of Sciences of the United States of America* 95:9448–9453.

Ma S, Pathak S, Trinh L, Lu R. 2008. Interferon regulatory factors 4 and 8 induce the expression of Ikaros and Aiolos to down-regulate pre-B-cell receptor and promote cell-cycle withdrawal in pre-B-cell development. *Blood* 111:1396–1403. DOI: 10.1182/blood-2007-08-110106.

Malinge S, Thiollier C, Chlon TM, Doré LC, Diebold L, Bluteau O, Mabialah V, Vainchenker W, Dessen P, Winandy S, Mercher T, Crispino JD. 2013. Ikaros inhibits megakaryopoiesis through functional interaction with GATA-1 and NOTCH signaling. *Blood* 121:2440–2451. DOI: 10.1182/BLOOD-2012-08-450627.

McKenna HJ, Stocking KL, Miller RE, Brasel K, De Smedt T, Maraskovsky E, Maliszewski CR, Lynch DH, Smith J, Pulendran B, Roux ER, Teepe M, Lyman SD, Peschon JJ. 2000. Mice lacking flt3 ligand have deficient hematopoiesis affecting hematopoietic progenitor cells, dendritic cells, and natural killer cells. *Blood* 95:3489–97.

Melo R, Longhini AL, Louzao Bigarella C, Ozello Baratti M, Traina F, Favaro P, De Melo Campos P, Olalla Saad ST. 2014. CXCR7 Is Highly Expressed in Acute Lymphoblastic Leukemia and Potentiates CXCR4 Response to CXCL12. *PLoS ONE* 9:e85926. DOI: 10.1371/journal.pone.0085926.

Miller JP, Izon D, DeMuth W, Gerstein R, Bhandoola A, Allman D. 2002. The earliest step in B lineage differentiation from common lymphoid progenitors is critically dependent upon interleukin 7. *The Journal of experimental medicine* 196:705–11. DOI: 10.1084/JEM.20020784.

Minegishi Y, Rohrer J, Coustan-Smith E, Lederman HM, Pappu R, Campana D, Chan AC, Conley ME. 1999. An essential role for BLNK in human B cell development. *Science (New York, N.Y.)* 286:1954–7. DOI: 10.1126/science.286.5446.1954.

Nagasawa T, Hirota S, Tachibana K, Takakura N, Nishikawa S, Kitamura Y, Yoshida N, Kikutani H, Kishimoto T. 1996. Defects of B-cell lymphopoiesis and BM myelopoiesis in mice lacking the CXC chemokine PBSF-SDF1. *Nature* 382:635–638.

Peled A, Kollet O, Ponomaryov T, Petit I, Franitza S, Grabovsky V, Slav MM, Nagler A, Lider O, Alon R, Zipori D, Lapidot T. 2000. The chemokine SDF-1 activates the integrins LFA-1, VLA-4, and VLA-5 on immature human CD34(+) cells: role in transendothelial/stromal migration and engraftment of NOD/SCID mice. *Blood* 95:3289–96.

Powers SE, Mandal M, Matsuda S, Miletic A V., Cato MH, Tanaka A, Rickert RC, Koyasu S, Clark MR. 2012. Subnuclear cyclin D3 compartments and the coordinated regulation of proliferation and immunoglobulin variable gene repression. *The Journal of Experimental Medicine* 209:2199–2213. DOI: 10.1084/jem.20120800.

Purizaca J, Contreras-Quiroz A, Dorantes-Acosta E, Vadillo E, Arriaga-Pizano L, Fuentes-Figueroa S, et al. 2013. Lymphoid progenitor cells from childhood acute lymphoblastic leukemia are functionally deficient and express high levels of the transcriptional repressor gfi-1. *Clinical & developmental immunology* 2013:349067. DOI: 10.1155/2013/349067.

Quintana-Bustamante O, Lan-Lan Smith S, Griessinger E, Reyal Y, Vargaftig J, Lister TA, Fitzgibbon J, Bonnet D. 2012. Overexpression of wild-type or mutants forms of CEBPA alter normal human hematopoiesis. *Leukemia* 26:1537–46. DOI: 10.1038/leu.2012.38.

Schebesta M, Pfeffer PL, Busslinger M. 2002. Control of pre-BCR signaling by Pax5-dependent activation of the BLNK gene. *Immunity* 17:473–85. DOI: 10.1016/S1074-7613(02)00418-1.

Schwartz R, Engel I, Fallahi-Sichani M, Petrie HT, Murre C. 2006. Gene expression patterns define novel roles for E47 in cell cycle progression, cytokine-mediated signaling, and T lineage development. *Proceedings of the National Academy of Sciences* 103:9976–9981. DOI: 10.1073/pnas.0603728103.

Shochat C, Tal N, Bandapalli OR, Palmi C, Ganmore I, te Kronnie G, Cario G, Cazzaniga G, Kulozik AE, Stanulla M, Schrappe M, Biondi A, Basso G, Bercovich D, Muckenthaler MU, Izraeli S. 2011. Gain-of-function mutations in interleukin-7 receptor-α (IL7R) in childhood acute lymphoblastic leukemias. *The Journal of Experimental Medicine* 208:901–908. DOI: 10.1084/jem.20110580.

Spooner CJ, Cheng JX, Pujadas E, Laslo P, Singh H. 2009. A Recurrent Network Involving the Transcription Factors PU.1 and Gfi1 Orchestrates Innate and Adaptive Immune Cell Fates. *Immunity* 31:576–586. DOI: 10.1016/j.immuni.2009.07.011.

Stein SJ, Baldwin AS. 2013. Deletion of the NF-κB subunit p65/RelA in the hematopoietic compartment leads to defects in hematopoietic stem cell function. *Blood* 121:5015–24. DOI: 10.1182/blood-2013-02-486142.

Tarnowski M, Liu R, Wysoczynski M, Ratajczak J, Kucia M, Ratajczak MZ. 2010. CXCR7: A new SDF-1-binding receptor in contrast to normal CD34+ progenitors is functional and is expressed at higher level in human malignant hematopoietic cells. *European Journal of Haematology* 85:472–483. DOI: 10.1111/j.1600-0609.2010.01531.x.

Teglund S, McKay C, Schuetz E, van Deursen JM, Stravopodis D, Wang D, Brown M, Bodner S, Grosveld G, Ihle JN. 1998. Stat5a and Stat5b proteins have essential and nonessential, or redundant, roles in cytokine responses. *Cell* 93:841–50. DOI: 10.1016/s0092-8674(00)81444-0.

Tian J, Li Z, Han Y, Jiang T, Song X, Jiang G. 2016. The progress of early growth response factor 1 and leukemia. *Intractable & Rare Diseases Research* 5:76–82. DOI: 10.5582/irdr.2015.01049.

Tothova Z, Kollipara R, Huntly BJ, Lee BH, Castrillon DH, Cullen DE, McDowell EP, Lazo-Kallanian S, Williams IR, Sears C, Armstrong SA, Passegué E, DePinho RA, Gilliland DG. 2007. FoxOs Are Critical Mediators of Hematopoietic Stem Cell Resistance to Physiologic Oxidative Stress. *Cell* 128:325–339. DOI: 10.1016/j.cell.2007.01.003.

Ueda Y, Yang K, Foster SJ, Kondo M, Kelsoe G. 2004. Inflammation Controls B Lymphopoiesis by Regulating Chemokine CXCL12 Expression. *The Journal of Experimental Medicine* 199:47–58. DOI: 10.1084/jem.20031104.

Urbánek P, Wang ZQ, Fetka I, Wagner EF, Busslinger M. 1994. Complete block of early B cell differentiation and altered patterning of the posterior midbrain in mice lacking Pax5/BSAP. *Cell* 79:901–12. DOI: 10.1016/0092-8674(94)90079-5.

Wang JH, Nichogiannopoulou A, Wu L, Sun L, Sharpe AH, Bigby M, Georgopoulos K. 1996. Selective defects in the development of the fetal and adult lymphoid system in mice with an Ikaros null mutation. *Immunity* 5:537–49.

Wierenga ATJ, Vellenga E, Schuringa JJ. 2010. Down-regulation of GATA1 uncouples STAT5-induced erythroid differentiation from stem/progenitor cell proliferation. *Blood* 115:4367–76. DOI: 10.1182/blood-2009-10-250894.

Yao Z, Cui Y, Watford WT, Bream JH, Yamaoka K, Hissong BD, Li D, Durum SK, Jiang Q, Bhandoola A, Hennighausen L, O’Shea JJ. 2006. Stat5a/b are essential for normal lymphoid development and differentiation. *Proceedings of the National Academy of Sciences of the United States of America* 103:1000–5. DOI: 10.1073/pnas.0507350103.

Zhang Z, Cotta C V, Stephan RP, deGuzman CG, Klug CA. 2003. Enforced expression of EBF in hematopoietic stem cells restricts lymphopoiesis to the B cell lineage. *The EMBO Journal* 22:4759–4769. DOI: 10.1093/emboj/cdg464.

Zhao F, Vilardi A, Neely RJ, Choi JK. 2001. Promotion of cell cycle progression by basic helix-loop-helix E2A. *Molecular and cellular biology* 21:6346–57. DOI: 10.1128/mcb.21.18.6346-6357.2001.

Zriwil A, Böiers C, Kristiansen TA, Wittmann L, Yuan J, Nerlov C, Sitnicka E, Jacobsen SEW. 2018. Direct role of FLT3 in regulation of early lymphoid progenitors. *British journal of haematology* 183:588–600. DOI: 10.1111/bjh.15578.
